# YY1 Knockdown Relieves the Differentiation Block and Restores Apoptosis in AML Cells

**DOI:** 10.3390/cancers15154010

**Published:** 2023-08-07

**Authors:** Nelida Ines Noguera, Serena Travaglini, Stefania Scalea, Caterina Catalanotto, Anna Reale, Michele Zampieri, Alessandra Zaza, Maria Rosaria Ricciardi, Daniela Francesca Angelini, Agostino Tafuri, Tiziana Ottone, Maria Teresa Voso, Giuseppe Zardo

**Affiliations:** 1 Department of Biomedicine and Prevention, Tor Vergata University, 00133 Rome, Italy; serenatravaglini@live.it (S.T.); tiziana.ottone@uniroma2.it (T.O.); voso@med.uniroma2.it (M.T.V.); 2 Unit of Neuro-Oncoematologia, Santa Lucia Foundation IRCCS, 00143 Rome, Italy; 3 Department of Experimental Medicine, Sapienza University, 00185 Rome, Italy; stefania.scalea@gmail.com; 4 Department of Molecular Medicine, Sapienza University, 00185 Rome, Italy; caterina.catalanotto@uniroma1.it (C.C.); anna.reale@uniroma1.it (A.R.); michele.zampieri@uniroma1.it (M.Z.); 5 Department of Medical and Surgical Sciences and Biotechnologies, Sapienza University, 00185 Rome, Italy; 6 Department of Clinical and Molecular Medicine, Sapienza University, 00185 Rome, Italy; mariarosaria.ricciardi@uniroma1.it (M.R.R.); agostino.tafuri@uniroma1.it (A.T.); 7 Laboratory of Neuro-Immunology and Cytofluorimetry, Santa Lucia Foundation IRCCS, 00143 Rome, Italy; df.angelini@hsantalucia.it

**Keywords:** YY1, acute myeloid leukemia, C/EBPs, RARα, all-trans retinoic acid, Polycomb proteins

## Abstract

**Simple Summary:**

Acute myeloid leukemia (AML) is characterized by the expansion of clonally derived hematopoietic precursors undergoing a partial or complete maturation block. De novo AML is characterized by recurrent cytogenetic alterations such as chromosomal translocations. However, in the recent past, it was shown that several somatic mutations and epigenetic alterations also contribute to AML onset and progression. Epigenetic mechanisms regulate the equilibrium between self-renewal and differentiation of hematopoietic stem cells and precursors. In this context, Polycomb-group (PcG) proteins regulate the expression of genes involved in cell-cycle regulation and differentiation, and aberrant expression and/or mutations of PcG genes have been shown to occur in hematopoietic neoplasms.

**Abstract:**

In this study we analyzed the expression of Yin and Yang 1 protein (YY1), a member of the noncanonical PcG complexes, in AML patient samples and AML cell lines and the effect of YY1 downregulation on the AML differentiation block. Our results show that YY1 is significantly overexpressed in AML patient samples and AML cell lines and that YY1 knockdown relieves the differentiation block. YY1 downregulation in two AML cell lines (HL-60 and OCI-AML3) and one AML patient sample restored the expression of members of the CEBP protein family, increased the expression of extrinsic growth factors/receptors and surface antigenic markers, induced morphological cell characteristics typical of myeloid differentiation, and sensitized cells to retinoic acid treatment and to apoptosis. Overall, our data show that YY1 is not a secondary regulator of myeloid differentiation but that, if overexpressed, it can play a predominant role in myeloid differentiation block.

## 1. Introduction

Acute myeloid leukemia (AML) is characterized by the expansion of clonally derived hematopoietic precursors undergoing a partial or a complete maturation block. De novo AML is characterized by recurrent cytogenetic alterations, such as chromosomal translocations [1], somatic mutations [2], and epigenetic alterations [3]. Epigenetic drivers of de novo AML include changes in DNA methylation, histone modifications, and post-transcriptional regulation of mRNAs by noncoding RNAs [4,5,6,7,8,9,10,11,12,13]. The relevance of epigenetic mechanisms in the pathogenesis of AML is so well established that they have become targets for therapy [14,15]. Epigenetic alterations may be the consequence of chromosomal rearrangements and mutations impacting the activity of specific epigenetic writers/readers/erasers. For instance, the fusion products PML-RARα, MLL-r, AML1-ETO, and MOZ-CBP induce epigenetic modifications that have a profound effect on chromatin architecture and the expression of targeted genes through the recruitment of histone deacetylase complexes, DNA methyltransferases, histone methyltransferases, and Polycomb repressor complexes [16,17,18,19,20,21,22]. Similarly, mutations of epigenetic writers/readers/erasers are frequent in AML. Loss of function or gain of function mutations of DNMT3A [23,24,25], EZH2 [26,27], TET2 [28,29], IDH1/2 [30], and ASXL1 [31,32], which may occur concurrently or be mutually exclusive, induce large-scale modifications of the epigenome, resulting in the deregulated expression of genes with a dominant role in normal and leukemic hematopoiesis.

In addition, epigenetic mechanisms regulate the equilibrium between self-renewal and differentiation of hematopoietic stem cells and precursors. In this context, Polycomb-group proteins (PcGs) regulate the expression of genes involved in cell-cycle regulation and differentiation, and aberrant expression and/or mutations of PcG genes have been shown to occur in hematopoietic neoplasms. PcG proteins form two complexes, named PRC1 and PRC2 [33,34]. PRC1 may be present in six different complexes, which are defined as canonical complexes (PRC1.2 and PRC1.4) depending on constitutive Polycomb ring finger proteins (PCGF1-6), and as noncanonical (PRC1.1, PRC1.3, PRC1.5, and PRC1.6) [33], whereas PRC2 has two main conformations, PRC2.1 and PRC2.2 [34]. PRC1 and PRC2 activity causes silencing of the targeted genes through their ability to catalyze posttranslational modifications of histone tails, histone H2A monoubiquitylation for PRC1, and histone H3 lysine 27 methylation for PRC2 [35,36,37,38]. The activity of the PcG complexes is counteracted by the trithorax (Trx) complexes, which, in contrast, deposit activating histone marks (H3K4 methylation) enabling gene transcription.

PcG and Trx proteins have been linked to several different hematological diseases and represent an interesting target for AML therapy [39]. BMI-1 is a main component of the PRC1 and shows oncogenic or tumor suppressor behavior in AML [40,41,42,43,44] and lymphoma [45]. CBX7 (PRC1) has a role in promoting T cell lymphomas and, in cooperation with MYC, in accelerating aggressive B cell lymphomagenesis through the regulation of the Ink4a/Arf locus (similarly to BMI-1) [46,47]. BCOR and BCORL1 products, components of the noncanonical PRC1 complex cooperating in recruiting it to CpG islands, are found frequently mutated in de novo and secondary AML, and often are downregulated in cytogenetically normal AML [48,49,50,51]. The heterozygous translocations of the mixed-lineage leukemia gene product (MLL-AF4; -AF9, -ENL, -AF10, and -ELL), a Trx complex component showing H3K4 histone methyltransferase activity, are found in a very high percentage of pediatric AML leukemias or acute lymphoblastic leukemias and determine the aberrant expression of the HOXA9 and MEIS1 genes, two master regulators of myeloid lineage [52,53,54]. EZH2, a core component of PRC2, is overexpressed in several distinct hematological disorders as well as in solid tumors. In addition, Stasik et al. showed that EZH2 gain- or loss-of-function mutations are quite frequent in de novo AML and seem to have a stage-specific role in AML [55]. SUZ12, EED, and JARID2 are other PRC2 components found to be mutated in myeloid diseases [56,57,58,59]. 

All these data underline the relevance and complexity of the roles of PcG and Trx complexes in the onset of the AML differentiation block. Thus, components of PcG and Trx complexes may undergo loss and gain of function mutations and chromosomal translocations capable of perturbing the sequential and coordinated expression of genes that control the hierarchical phenomenon of hematopoiesis. However, the mechanism of their recruitment to targeted genes has been a longstanding topic of study because mammalian PcG and Trx proteins lack recognition and binding sequences on DNA. In Drosophila melanogaster, Pleiohomeotic (Pho) protein, a zinc finger DNA binding protein, is responsible for recruiting PcG proteins to the PREs of target genes [60,61]. The mammalian homolog of Pho is Yin Yang 1 (YY1) protein [59], a Polycomb-group protein with sequence-specific DNA binding [62], which is considered a candidate targeting factor [63] able to recruit PcG complexes to targeted genes [64,65,66,67]. YY1 interacts with the PcG proteins EED and BMI1 in separate complexes and colocalizes in the trunks of E12.5 mouse embryos upstream of the repressed HOXA5 and HOXC8 genes [68]. Moreover, YY1 directly interacts with RYBP and YAF2, which are components of noncanonical PRC1 complexes, suggesting that they may link YY1 with the PcG system. YY1 protein may act as an activator or repressor of transcription, depending on posttranslational modifications, binding partners, and cellular context [69,70,71]. 

YY1 mutations or altered expression frequently occurs in disease. YY1 haploinsufficiency has been reported in a case of Gabriele–de Vries syndrome [72] and in neurodevelopmental disorders [73] in addition to neurodegenerative diseases, such as Alzheimer’s and Parkinson’s [74,75]. The role of YY1 in cancer is highly dependent on the expression level, and it may act as tumor repressor or oncogene [76]. YY1 promotes tumor growth in melanoma as a conditional ablation of one YY1 allele; in a melanoma mouse model, it prevents tumorigenesis [77]. YY1, through several distinct mechanisms, promotes the proliferation of glioma tumor cells [78,79]. YY1 overexpression stimulates cell proliferation and migration in laryngeal cancer cells by directly inhibiting MYCT1 [80]. Kaufhold et al. identified YY1 as a regulatory factor involved in cancer stem cell (CSC) maintenance across 17 different cancer types [81]. YY1 interferes with control of the cell cycle and apoptosis. YY1 overexpression induces cell progression into the S phase, overcoming Rb-induced cell cycle arrest at the G1/S checkpoint [82]. YY1 negatively regulates p53-dependent apoptosis, p16-dependent senescence [83,84], and activates the c-Myc promoter [85]. 

Regarding the role of YY1 in hematopoiesis, Pan et al. [86] showed that transplantation of bone marrow progenitors ectopically expressing the YY1 gene increased LSK (Lin(-)Sca-1(+)c-Kit(+)) and the LT-HSC (long-term hematopoietic stem cell) populations in mice, highlighting a role for YY1 in enhancing the self-renewal potential of these cells. Moreover, the levels of short-term (ST)-HSCs, multipotent progenitors, myeloid progenitors, monocytes, and neutrophils were also increased, suggesting that YY1 mediates the multipotency and differentiation potential of HSCs [87]. On the other hand, the analyses of the expression levels of PcG genes, including YY1, in bone marrow samples from 126 AML patients vs. 20 healthy donors showed consistent YY1 overexpression [88]. Erkeland et al. [89] showed that YY1 protein levels were high in 32D parental cells maintained in interleukin-3-containing medium, but they dropped when the cells were induced to differentiate with granulocyte-colony-stimulating factor (G-CSF). Furthermore, G-CSF-induced neutrophilic differentiation was reduced in 32D cell transfectants ectopically expressing YY1. In primary bone marrow cells, YY1 overexpression blocked the growth of CFU-GM colonies. In addition, YY1 is associated with the antitumor therapy resistance of leukemia cells [90]. Collectively, these data suggest a possible role for perturbed expression of YY1 in the development of AML through interference with the myeloid differentiation program.

Here, we investigate the overexpression of YY1 in primary AML samples and use knockdown strategies to elucidate the role of YY1 in myeloid differentiation and the development of AML. The results indicate that targeting YY1 relieves the differentiation block in AML and renders cells more sensitive to conventional treatment with the differentiating agent all-trans retinoic acid (ATRA).

## 2. Materials and Methods

### 2.1. Reagents

All-trans retinoic acid (ATRA) was purchased from Sigma-Aldrich (CAS N: 302-79-4; Milan, Italy) and used at a concentration of 1 µM for up to 96 h. Bortezomib was purchased from Selleckchem (PS-341; Houston, TX, USA) and used at a concentration of 2.5 nm for up to 96 h.

### 2.2. Ethics Statement

The study was approved by the ethical committee of the University of Rome Tor Vergata (Study Protocol 171/19).

### 2.3. Human Samples and AML Cell Lines 

Immature CD34+ and mononuclear CD34− cell fractions were purified from the cord blood of four healthy donors with immunomagnetic column separation (Miltenyi Biotec Inc.; Auburn, CA, USA; and STEMCELL Technologies; Cambridge, MA, USA). Cord blood was provided by the UOS Regional Bank of Cord Blood. Cells were labeled with human anti-CD34-APC (Miltenyi Biotech Inc., Gaithersburg, Maryland, MD, USA) and sorted on the FACS Aria III (Becton Dickinson, BD Biosciences; Franklin Lakes, NJ, USA). 

Acute myeloid leukemia (AML) samples (*n* = 24) were obtained from the peripheral blood or bone marrow of newly diagnosed leukemia patients showing more than 60% leukemic infiltration (see Supplementary Table S1 for sample features).

The HL-60 cell line was cultured in RPMI 1640 (Euroclone; Pero, MI, Italy) supplemented with 10% fetal bovine serum (FBS) (Gibco; Thermo Fisher Scientific; Waltham, MA, USA) and 100 U/mL penicillin and 100 µg/mL streptomycin (Euroclone; Pero, MI, Italy). The OCI-AML3 cell line, an AML-M4-derived cell line carrying an NPM1 gene mutation (type A) and the DNMT3A R882C mutation, were kindly provided by Emanuela Colombo, the European Institute of Oncology, Milan, Italy. The OCI-AML3 cells were cultured in RPMI 1640, 10% FBS, 100 U/mL penicillin, and 100 µg/mL streptomycin. ML-2 and ME-1 cell lines were cultured in RPMI 1640 supplemented with 20% FBS and 1% penicillin–streptomycin. The AML-193 cell line was cultured in Iscove’s MDM (Biowest, Nuaillé, Francia) supplemented with 10% FBS, 1% penicillin–streptomycin, and 20% conditioned medium from the cell line 5637 (DSMZ ACC 35)(Braunschweig, Germany). Cultures were maintained at 37°C in a 5% CO_2_ humidified incubator.

### 2.4. Plasmid Constructs, Lentiviral Infection, and Cell Transfection

Knockdown of YY1 in the HL-60 cell line was performed by cloning a short hairpin RNA targeting YY1 (AgeI-EcoRI) into the Tet-pLKO.1 puro vector of the tetracycline inducible system. The following shRNA sequences were used: 

sh-YY1:

5′-CCGGATGACAGGAAAGAAACTTCCTCTCGAGAGGAAGTTTCTTTCCTGTCATTTTTT-3′; 

and sh-scrambled control sequence: 

5′-CCGGGCAACAAGATGAAGAGCACCAACTCGAGTTGGTGCTCTTCATCTTGTTGCTTTTT-3′. 

Plasmids were verified by sequencing. Lentivirus was produced in HEK293T cells transfected with Tet-pLKO.1 puro-sh-YY1 and Tet-pLKO.1 puro-sh-control constructs (Addagene, Watertown, MA, USA) with a third-generation lentiviral system. The supernatant containing lentivirus particles was collected at 48 and 72 h post-transfection, concentrated by ultracentrifugation, and resuspended in PBS-1% BSA. HL-60 cells were infected twice with concentrated lentiviral particles in medium supplemented with polybrene (8 µg/mL) (Sigma Aldrich, St Luis, MO, USA) and then selected in puromycin (0.25 µg/mL) (Sigma Aldrich, St Luis, MO, USA). After selection, infected HL-60 cells were maintained in culture with a low puromycin concentration of 0.125 µg/mL and treated with doxycycline (300 ng/mL) (Sigma Aldrich, St Luis, MO, USA) to induce shRNA expression.

For siRNA-mediated gene knockdown in OCI-AML3, OCI-AML3 cells (1 × 106 cells/well) were seeded into 12-well plates and transfected with 100 nM of siRNA targeting YY1 (cod. 0007774146 siRNA ID s14960 and s14958, Ambion, Life Technologies, Thermo Fisher Scientific, Waltham, MA, USA) or control siRNA (D-001810-01-05) with Lipofectamine RNAiMAX (4 µL/well; Thermo Fisher Scientific, Waltham, MA, USA). Silencing was performed at 0 and 24 h, and the cells were treated with ATRA (1 µM) at 48 h. Cells were collected at 48 h and 96 h, according to the manufacturer’s protocol.

### 2.5. Protein Extraction and Western Blot Analysis

Cells were lysed with RIPA lysis buffer containing 1× proteinase inhibitors, and proteins were quantified with the Bradford assay. Equal amounts of protein (20–50 µg) were denatured, separated on SDS polyacrylamide gels, and blotted onto Bio-Rad Immun-Blot PVDF membranes. The following primary antibodies and dilutions were used for immunoblotting detection: rabbit anti-human YY1 (1:600, sc-1703, Santa-Cruz Biotechnology; Dallas, TX, USA); rabbit anti-human PARP1 (1:4000, ALX-210-302-R100, Life Sciences); rabbit anti-human BAX (1:200, #2772, Cell Signaling Technology; Danvers, MA, USA); rabbit anti-human caspase-3 (1:800, #9662, Cell Signaling Technology); rabbit anti-human C/EBPα (1:800, EP709Y-ab40764, Abcam; Cambridge, MA, USA); rabbit anti-human C/EBPδ (1:500, EPR23518-259-ab245214, Abcam); and rabbit anti-human C/EBPε (0.6 μg/mL, ab246861, Abcam). Mouse anti-human α-tubulin (1:5000, #3873, Cell Signaling Technology) and mouse anti-human β-actin (1:10,000, #3700, Cell Signaling Technology) were used for normalization of the samples analyzed. The following horseradish peroxidase-conjugated secondary antibodies were used: anti-mouse polyclonal IgG (Goat anti-Mouse IgG #31430, Invitrogen; Thermo Fisher Scientific) and anti-rabbit polyclonal IgG (Goat anti-Rabbit IgG #31460, Invitrogen; Thermo Fisher Scientific). Chemiluminescence detection was performed with ECL (Amersham Biosciences; Thermo Fisher Scientific, Waltham, MA, USA), and images were captured with the Bio-Rad ChemiDoc XRS+ imaging system. Protein signal intensities were analyzed by densitometric scanning with Quantity One software (Bio-Rad Laboratories, Hercules, CA, USA), and normalized with the loading control β-actin or tubulin. The original western blot figures could be found in File S1.

### 2.6. RNA Isolated and Analysis

Total RNA was isolated from cells with TRIzol (Invitrogen; Thermo Fisher Scientific; Waltham, MA, USA). cDNA was synthesized from total RNA (1 µg) with the High-Capacity RNA-to-cDNA Kit (Applied Biosystems; Thermo Fisher Scientific), and real-time quantitative RT-PCR was performed to determine expression levels for *YY1*, *HOXA2*, *HOXD13*, *C/EBPα*, *C/EBPδ*, *C/EBPε*, *CD11b*, *CD14*, *GM-CSFr*, *CSF1*, *G-CSFr*, *CSF2*, *M-CSFr*, *CSF3*, *RARα*, and *GAPDH*. The sequences of the primer pairs used are listed in supplementary methods. All reactions were performed in triplicate on total RNA isolated from three independent cell cultures. Real-time PCR was performed with the SYBR Green dye detection method. Ct values obtained for genes in the samples were normalized with Ct values from GAPDH and calculated following the 2^−ΔΔCT^ or 2^−ΔCT^ method, alternatively.

### 2.7. Chromatin Immunoprecipitation (ChIp)

Chromatin immunoprecipitations were performed on lysates prepared from HL-60 cells and using rabbit anti-human YY1 (sc-1703, Santa-Cruz Biotechnology) following a standard protocol. Rabbit anti-human IgG antibodies (Merck Millipore; Burlington, MA, USA) were used as immunoprecipitation controls. Genomic regions in 5′ promoter sites, within 1 kb of the putative TSS, of *RARα*, *C/EBPα*, *C/EBPδ*, *C/EBPε*, and *GAPDH* genes were amplified from immunoprecipitated DNAs. The sequences of the primer pairs used are listed in supplementary methods. All primer pairs were designed with Primer Express Version 3.0 software (Applied Biosystems; Foster City, CA, USA). qRT-PCR was performed in triplicate with SYBR Green. Values obtained for the DNA in each immunoprecipitated sample were quantified relative to the respective input and calculated following the 2^−ΔCT^ method.

### 2.8. Immunophenotypic Analysis

Immunophenotyping was performed with direct immunofluorescence staining of cells: for the HL-60 cell line, APC-conjugated mouse anti-human CD11b (clone ICRF44) and CD14 (clone M5E2; Becton Dickinson Pharmingen); and for the OCI-AML3 cell line, APC-conjugated mouse anti-human CD11b (clone ICRF44, Beckton Dickinson Pharmingen) and CD14 (clone M5E2, Beckton Dickinson Pharmingen). A minimum of 50,000 events was recorded for each sample on a FACSCantoII flow cytometer (BD Biosciences) for HL-60 and on a CytoFLEX flow cytometer (Beckman Coulter, Brea, CA, USA) for OCI-AML3. Apoptotic cell death in HL60 cells was evaluated with the APC Annexin-V Apoptosis Detection Kit with PI (BioLegend, San Diego, CA, USA) according to the manufacturer’s instructions on a FACSCantoII flow cytometer (BD Biosciences, Franklin Lakes, NJ, USA), and in OCI-AML3 cells, with Annexin V staining and the ‘Live/Dead’ assay on a CytoFLEX flow cytometer (Beckman Coulter; Brea, CA, USA). Flow cytometric analysis was performed with FlowJo Flow Cytometric Analysis software (TreeStar; Ashland, OR, USA). Cells labeled with a single fluorochrome were used as controls to adjust the compensation. 

### 2.9. Morphological Analysis

HL-60 and OCI-AML3 cell morphology was evaluated using staining cytospin (8 min at 900 rpm) with Wright-Giemsa stains, according to the manufacturer’s instructions. Images were captured with a Nikon Eclipse 80i upright microscope. 

### 2.10. Statistical Analysis 

All data are expressed as mean ± DS, and the ± DS are represented by error bars. The statistical significance was calculated with a one-tailed Mann–Whitney non-parametric test with GraphPad Prism 5, and a *p*-value ≤ 0.05 was considered as significant. The experiments were performed at least three times in duplicate unless otherwise stated.

## 3. Results

### 3.1. An Inhibitor of NF-kB Activation, Bortezomib, Downregulates YY1 in AML Cells

**Figure 1:**
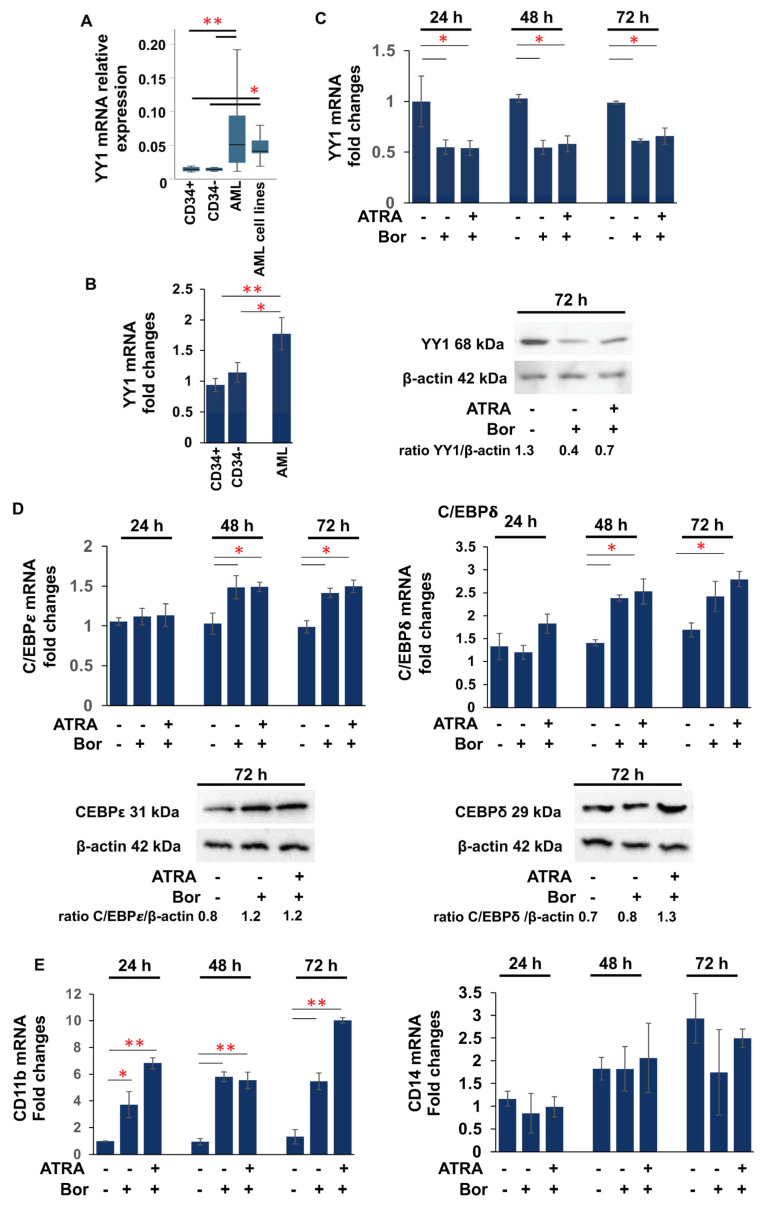
Bortezomib downregulates YY1 expression in patient AML samples. (**A**) qRT-PCR showing *YY1* mRNA relative expression in 24 AML human samples and 5 AML cell lines compared with immature CD34+ and mature CD34- cells. (**B**) qRT-PCR showing *YY1* mRNA relative expression in the acute myeloid leukemia (AML) human sample compared with immature CD34+ and mature CD34- cells, successively treated with Bortezomib (Bor) alone or in association with 1 μM ATRA up to 72 h. (**C**) qRT-PCR and Western blot showing *YY1* mRNA relative expression and protein amount in the AML samples, untreated and Bor- and Bor+ATRA-treated. (**D**) qRT-PCR and Western blot showing *YY1* mRNA relative expression and protein amount of C/EBPε and C/EBPδ in the AML samples, untreated and Bor- and Bor+ATRA-treated. (**E**) qRT-PCR showing *YY1* mRNA relative expression of *CD11b* and *CD14* in the AML samples, untreated and Bor- and Bor+ATRA-treated. In qRT-PCR, the expression levels were normalized to *GAPDH* levels. Each sample was analyzed in triplicate. Statistical analysis was performed with the nonparametric Mann–Whitney test; * *p*-value ≤ 0.05; ** *p*-value ≤ 0.01. Error bars: SD, standard deviation.

The proteasome inhibitor Bortezomib, approved by the FDA for clinical use in multiple myeloma and in AML clinical trials [91,92], has been shown to inhibit NF-kB activation [93]. About 40% of AML patients exhibit increased activity of the NF-kB signaling pathway, and it is constitutively active in CD34+/CD38- blasts from M1, M2, M4, and M5 AML patients [94,95]. YY1 is a target of NF-kB activation [96,97]. Therefore, to further elucidate the role of YY1 in hematopoietic differentiation and the development of AML, we investigated whether Bortezomib decreased the expression of YY1 in AML cells. We first analyzed YY1 expression in 24 AML samples (Supplementary Table S1) and 5 AML cell lines (HL-60, AML-193, ML-2, ME-1, and OCI-AML3) [88,89]. qRT-PCR analysis revealed a statistically significant increase in YY1 mRNA levels in the AML patient samples and AML cell lines compared with four immature CD34+ cells and four CD34- mononuclear mature cell samples purified from umbilical cord blood of four healthy donors (Figure 1A). Next, we treated cells from a YY1 high-expressing AML sample (Figure 1B) with 2.5 nM Bortezomib and analyzed YY1 mRNA and protein levels over 72 h at 24 h intervals (Figure 1C). Bortezomib significantly reduced YY1 expression as early as 24 h. However, co-treatment with the vitamin ATRA (1 μM) did not lead to a further decrease in YY1 in Bortezomib+ATRA-treated AML cells (Figure 1C). Indeed, Bortezomib treatment led to an increase in the mRNA and protein levels of transcription factors C/EBPε and C/EBPδ (CCAAT/enhancer binding protein ε, δ) (Figure 1D), which are directly involved in granulocytic terminal differentiation, whereas no difference in *C/EBPα* expression was observed.

Remarkably, Bortezomib caused an increase in the expression of the granulocytic surface antigen differentiation marker CD11b, whereas no change in expression was observed for the monocytic surface antigen CD14 (Figure 1E). 

### 3.2. YY1 Knockdown Induces Expression of Myeloid Differentiation Markers

**Figure 2:**
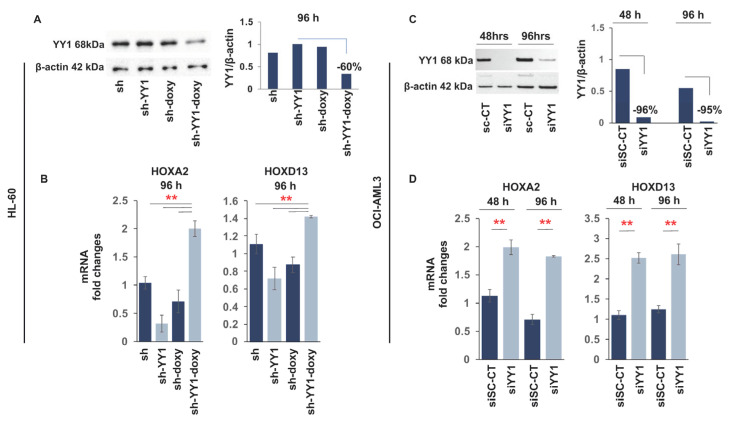
YY1 knockdown in HL-60 and OCI-AML3 cell lines induces expression of HOXA2 and HOXD13. (**A**) Western blot and quantitative analysis showing YY1 knockdown in HL-60 cells with shRNA. YY1 knockdown was achieved with lentiviral infection of a short hairpin RNA targeting YY1 cloned into a Tet-pLKO.1 puro vector. Infected HL-60 cells were maintained in culture with a low puromycin concentration and treated with doxycycline (300 ng/mL) to induce shRNA expression. sh: HL-60 cells carrying a short hairpin scrambled control sequence; sh-YY1: HL-60 cells carrying a short hairpin RNA targeting YY1; sh-doxy: HL-60 cells treated with doxycycline to induce scrambled shRNA expression; and sh-YY1-doxy: HL-60 cells treated with doxycycline to induce YY1 shRNA expression. (**B**) qRT-PCR measuring the relative expression of HOXA2 and HOXD13 mRNAs in HL-60 cells with YY1 knockdown. (**C**) Western blot and quantitative analysis showing YY1 knockdown in the OCI-AML3 cell line with siRNA. YY1 knockdown was achieved with transfection of an siRNA targeting YY1 (siYY1). YY1 expression in siYY1 cells was compared with the expression in OCI-AML3 cells transfected with a control siRNA (siSc-CT) sequence. Cells were collected after 48 h and 96 h following transfection. (**D**) qRT-PCR measuring the relative expression of HOXA2 and HOXD13 mRNAs in OCI-AML3 cells with YY1 knockdown. ** *p*-value ≤ 0.01. Error bars: SD, standard deviation.

Based on these results and data in the literature [86,87,88,89], we investigated the possibility that YY1 overexpression could interfere with the myeloid differentiation program in AML cell lines. YY1 protein levels were downregulated in the HL-60 cell line (FAB M2) with a doxycycline inducible lentiviral vector expressing a short hairpin against YY1 mRNA, and in the OCI-AML3 (FAB M4) cell line, with the transfection of a small-interfering RNA targeting YY1. Both knockdown approaches caused a significant reduction in YY1 protein in these cell lines, at 120 h of doxycycline induction (300 ng/mL) in HL-60-sh-YY1-doxy and at 48 h and 96 h in OCI-AML3-siYY1 (Figure 2A,B). Furthermore, the mRNA expression of HOXA2 and HOXD13 genes increased in response to YY1 knockdown, demonstrating that knockdown of YY1 in these cells led to a previously characterized functional response of the downregulation of YY1 protein [96] (Figure 2C,D).

**Figure 3:**
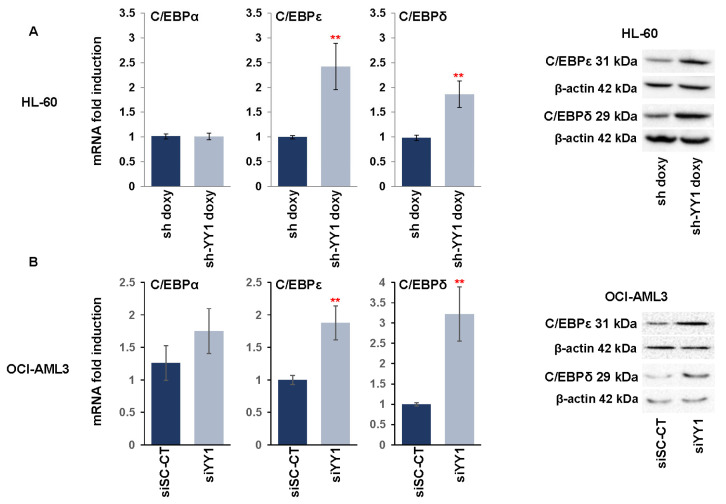
YY1 knockdown increases expression of C/EBP family transcription factors. (**A**) qRT-PCR and Western blots measuring CEBPα, CEBPε, and CEBPδ mRNA and protein expression levels performed in doxycycline-induced HL-60-sh-YY1-doxy cells compared with doxycycline-induced HL-60 control sh-doxy cells. mRNA expression levels were normalized to GAPDH levels and expressed as fold induction relative to the values of the control sh-doxy cells (=1). (**B**) qRT-PCR and Western blots assessing CEBPα, CEBPε, and CEBPδ expression in OCI-AML3 siYY1 cells compared with OCI-AML3 siSc-CT cells. mRNA expression levels were normalized to GAPDH levels and expressed as fold induction relative to the value of the control cells (=1). Each qRT-PCR amplification was performed in triplicate. Statistical analysis was performed with the nonparametric Mann–Whitney test. ** *p*-value ≤ 0.01. Error bars: SD, standard deviation.

We next investigated the effect of YY1 knockdown on C/EBP family members α, ε, and δ, which have an important role in normal hematopoiesis, mainly in the promotion of myeloid differentiation, and which are decreased in expression or frequently mutated in AML [98,99,100,101]. Knockdown of YY1 in the HL-60-sh-YY1-doxy and OCI-AML3-siYY1 cell lines caused a statistically significant increase in C/EBPε and C/EBPδ expression, both at the transcriptional and protein levels compared with control cells (Figure 3A,B). 

Finally, we assessed the expression levels of growth factors involved in myeloid differentiation, including colony-stimulating factor 2 (CSF2), 3 (CSF3), and 1 (CSF1), and the receptors they bind to, granulocyte-macrophage-colony-stimulating factor receptor (GM-CSFr), granulocyte-colony-stimulating factor receptor (G-CSFr), and macrophage-colony-stimulating factor receptor (M-CSFr). We found that while the levels in growth factors did not change, GM-CSFr was significantly upregulated in both HL-60-sh-YY1-doxy and OCI-AML3-siYY1, while G-CSFr was significantly upregulated only in HL-60-sh-YY1-doxy (Supplementary Figure S1).

### 3.3. YY1 Knockdown Increases Expression of C/EBP Family Proteins and Myeloid Growth Factors/Receptors in ATRA-Treated HL-60 and OCI-AML3 Cell Lines

**Figure 4:**
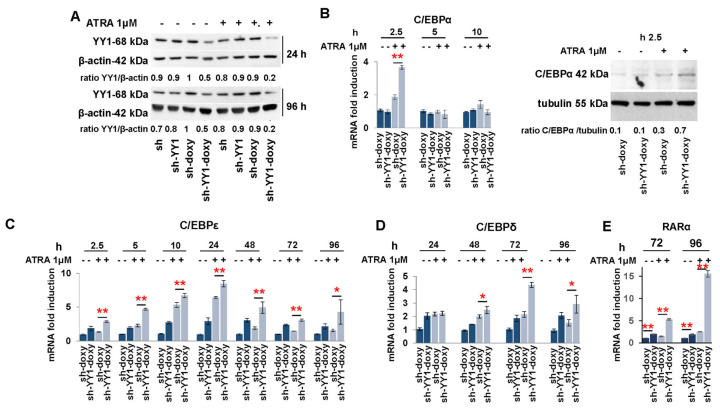
YY1 knockdown increases the sensitivity of HL-60 cells to ATRA treatment. (**A**) Western blot analysis of YY1 protein levels in sh and sh-YY1 cells, induced with doxycycline or not, collected at 24 h and 96 h of ATRA treatment. (**B**) qRT-PCR and Western blot to quantify *C/EBPα* in sh-YY1-doxy and control sh-doxy cells treated or untreated with 1 μM ATRA for the indicated time points. (**C**–**E**) qRT-PCR to quantify *C/EBPε, C/EBPδ,* and *RARα* in sh-YY1-doxy and control sh-doxy cells treated or untreated with 1 μM ATRA for the indicated time points. Expression levels were normalized to *GAPDH* levels and expressed as fold induction to the value for sh-doxy cells (=1). Each qRT-PCR amplification was performed in triplicate. Statistical analysis was performed in triplicate using with the nonparametric Mann–Whitney test. * *p* value ≤ 0.05; ** *p* value ≤ 0.01. Error bars: SD, standard deviation.

The knockdown of YY1 protein in the AML patient sample and in the HL-60 and OCI-AML3 cell lines led to an increase in the expression of genes associated with myeloid differentiation, including myeloid transcription factors (C/EBPε, C/EBPδ), myeloid growth factors receptors (GM-CSFr, G-CSFr), and the granulocytic differentiation marker (CD11b). The HL-60 cell line is a model for granulocytic differentiation when treated with ATRA [102], although this cell line does not carry the PML/RARα translocation. The OCI-AML3 cell line carries the nucleophosmin gene mutation (NPM1*mut*), which is found in approximately 30% of adult leukemia cases [103]. Treatment of NPM1*mut* AMLs with ATRA, or in combination with ATO or chemotherapy, has been shown to increase apoptosis and improve the outcomes of elderly patients [104,105,106]. Therefore, we asked if knockdown of YY1 increased the sensitivity of the HL-60 and OCI-AML3 cell lines to ATRA treatment. HL-60-sh and -sh-doxy, -sh-YY1-doxy following doxycycline induction, and OCI-AML3 siSC-CT and -siYY1 after transfection were collected during treatment with 1 μM ATRA over 96 h at 24 h intervals. YY1 protein levels were efficiently downregulated throughout the ATRA exposure time in HL-60-sh-YY1-doxy relative to control cells (Figure 4A). Exposure to 1 μM ATRA led to a statistically significant increase in expression of *C/EBPα*, *C/EBPε,* and *C/EBPδ* in HL-60-sh-YY1-doxy relative to the control HL-60-sh-doxy at all time points (Figure 4B–D). However, *C/EBPα* showed increased expression as early as 2.5 h after exposure to ATRA, also confirmed with Western blot (Figure 4B), while C/EBPδ did not show an increase until the 24 h time point for ATRA exposure (Figure 4D). 

**Figure 5:**
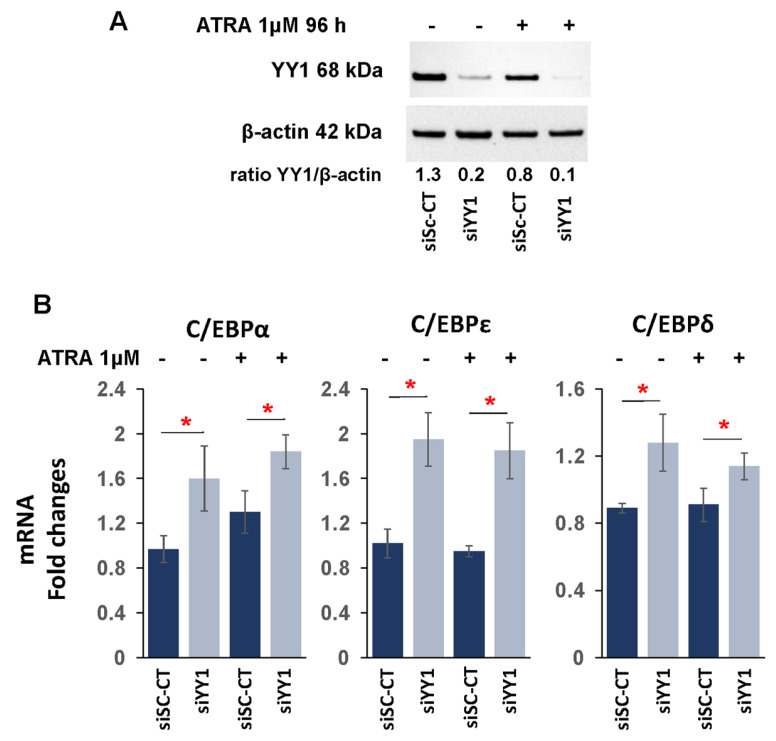
YY1 knockdown does not sensitize the OCI-AML3 cell line to ATRA exposure. (**A**) Western blot analysis of YY1 protein level in siSc-CT and siYY1 cells, treated with ATRA or not, for 96 h. (**B**) qRT-PCR assessing C/EBPα, C/EBPɛ, and C/EBP-δ mRNA relative expression in siSc-CT and siYY1 cells, treated with ATRA or not, for 96 h. Each qRT-PCR amplification was performed in triplicate. Statistical analysis was performed with the nonparametric Mann–Whitney test. * *p*-value ≤ 0.05; Error bars: SD, standard deviation.

Expression of the RARα cytoplasmic ATRA receptor was also increased in HL-60-sh-YY1-doxy, and this increase was enhanced several fold with ATRA treatment (Figure 4E). In addition, CSF1, CSF3, GMCSF-r, GCSF-r, and MCSF-r were statistically increased in ATRA-treated HL-60-sh-YY1-doxy relative to ATRA-treated HL-60-sh-doxy and untreated HL-60-sh-YY1-doxy (Supplementary Figure S2). Expression of C/EBPα, C/EBPε, and C/EBPδ was also increased in ATRA-treated OCI-AML3-siYY1 relative to ATRA-treated OCI-AML3-siSc-CT. YY1 protein levels were efficiently downregulated throughout the ATRA exposure time also in OCI-AML3-siYY1 (Figure 5A); however, this increase was not significantly greater than the expression of these genes in untreated OCI-AML3-siYY1 (Figure 5B).

### 3.4. YY1 binds C/EBPα, C/EBPε, C/EBPδ, and RARα Gene Promoters

**Figure 6:**
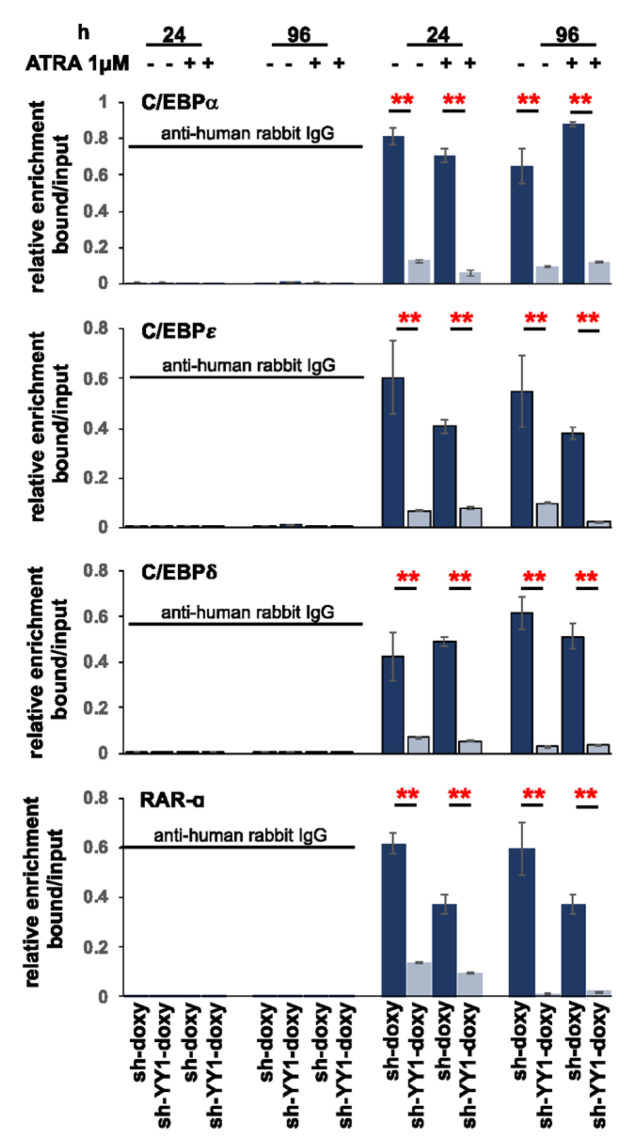
YY1 binds 5′ promoter regions of C/EBPα, C/EBPε, C/EBPδ, and RARα genes. Chromatin immunoprecipitation showing the YY1 occupancy at the 5′ promoter regions of C/EBPα, C/EBPε, C/EBPδ, and RARα genes in 1 μM ATRA-treated or -untreated HL-60-YY1-sh-doxy and -sh-YY1-doxy cells. Experiments were conducted with two different preparations of HL-60 cells. Each qRT-PCR amplification was executed in triplicate three times, and the results of the two independent experiments were collected and used for calculation (two different experiments and a total of eighteen measurements for each sample). Rabbit anti-human IgG was used as the immunoprecipitation control. Statistical analyses were performed with the nonparametric Mann–Whitney test. ** *p*-value ≤ 0.01. Error bars: SD, standard deviation.

Knockdown of YY1 determined an increased expression in some C/EBP protein family members in both cell lines and RARα in HL-60 cells. In these cells, YY1 could act as a transcriptional repressor protein of these genes, and loss of the protein may relieve the promoter regions of the C/EBPs and RARα genes of YY1 transcriptional repression. The Jaspar core 2022 database (consultable on the UCSC genome browser) predicted the presence of putative YY1 binding sites in the 5′ promoter regions of C/EBPα, C/EBPε, C/EBPδ, and RARα. Therefore, we performed chromatin immunoprecipitation (ChIp) with lysates prepared from HL-60-sh-YY1-doxy and -sh-doxy cells to determine YY1 occupancy of the 5′ promoter regions of the C/EBP and RARα genes. Immunoprecipitations were performed on lysates prepared from HL-60-sh-YY1-doxy and -sh-doxy cells induced with doxycycline for 96 h and treated or untreated with ATRA for an additional 96 h. ChIp data showed reduced binding of the 5′ promoter regions of C/EBPα, C/EBPε, C/EBPδ, and RARα in the HL-60-sh-YY1-doxy cells, thus demonstrating that YY1 bound to these putative sites (Figure 6). Consequently, the increased expression of these genes in HL-60 (sh-YY1-doxy) relative to HL-60 (sh-doxy) cells might be due to reduced binding of YY1 to their promoter regions, and thus, the loss of its repressive effect on the transcription of these genes. In the case of C/EBPα, ChIp data confirmed reduced YY1 binding at the 5′ promoter region in sh-YY1-doxy cells; however, this was not accompanied by an increase in the C/EBPα expression, apparently in contradiction with the increased expression observed when the same cells were exposed to ATRA (Figure 4B). A possible explanation might be that the C/EBPα promoter, following YY1 displacement, will became available to the binding of an ATRA-induced transcription factor.

### 3.5. YY1 Knockdown Enhances Expression of Myeloid Differentiation Markers and Cell Morphological Differentiation Features

**Figure 7:**
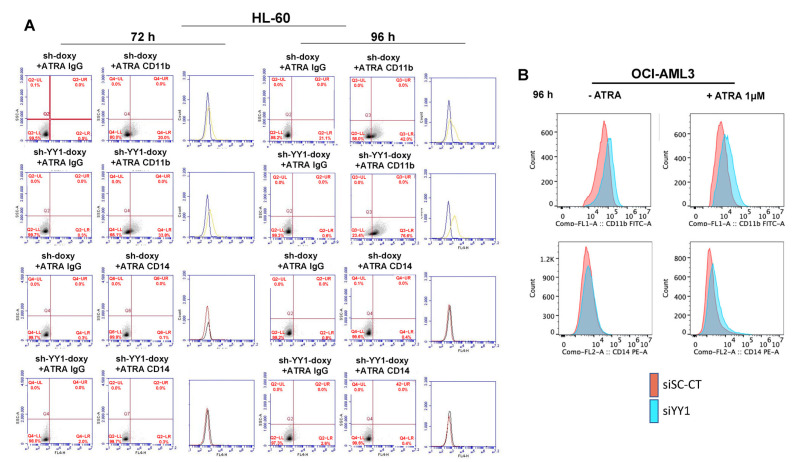
YY1 knockdown induces differentiation immunophenotype in 1 μM ATRA-treated or -untreated HL-60 and OCI-AML3 cells with YY1 knockdown. (**A**) Flow cytometry plots showing the detection of CD11b and CD14 surface differentiation markers in HL-60-sh-doxy and sh-YY1-doxy cells treated with 1 μM ATRA for 72 h and 96 h. (**B**) Flow cytometry plots showing the detection of CD11b and CD14 surface differentiation markers in OCI-AML3 siSc-CT and siYY1 cells treated with 1 μM ATRA for 96 h.

To investigate the relationship between YY1 knockdown and myeloid differentiation, we monitored the expression of surface adhesion molecules, granulocytic CD11b and monocytic CD14 [107], and changes in the cell morphology of HL-60-sh-YY1-doxy and -sh-doxy and OCI-AML3-siYY1 and -siSC-CT cells, treated or untreated with 1 μM ATRA. Treatment with 1 μM ATRA increased expression of the granulocytic CD11b marker in HL-60-sh-YY1-doxy at 72 h and 96 h relative to controls (33.8% vs. 20% and 76.6% vs. 42%, ATRA-treated HL-60-sh-YY1-doxy vs. HL-60-sh-doxy, at 72 h and 96 h, respectively). The expression of the monocytic CD14 marker remained unchanged between the samples (Figure 7A). 

CD11b showed a slight increase in ATRA-untreated OCI-AML3-siYY1 cells at 96 h after YY1 siRNA transfection (+13% relative to the control OCI-AML3-siSC-CT cells). The effect of the loss of YY1 was more robust in the ATRA-treated cells, with +34% C11b positive OCI-AML3-siYY1 cells compared with OCI-AML3-siSC-CT. CD14 increased by 16% in ATRA-untreated OCI-AML3-siYY1 cells compared with OCI-AML3-siSC-CT, and by 28% in ATRA-treated OCI-AML3-siYY1 compared with ATRA-treated OCI-AML3-siSC-CT (Figure 7B). 

**Figure 8:**
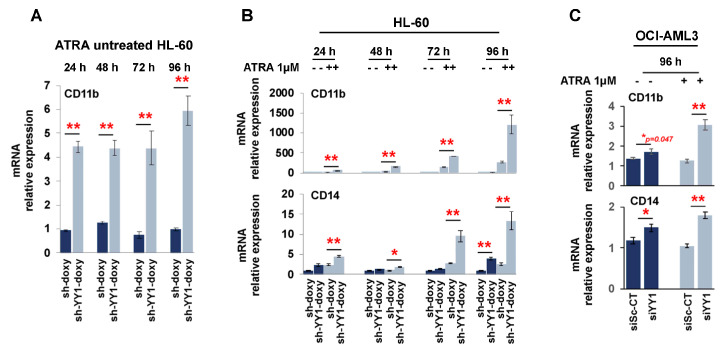
YY1 knockdown promotes expression of surface differentiation markers CD11b and CD14 in HL-60 and OCI-AML3 cells treated or untreated with 1 μM ATRA. (**A**) Relative mRNA expression of CD11b and CD14 surface differentiation markers in ATRA-untreated HL-60-sh-doxy and sh-doxy-YY1 cells over 96 h at 24 h intervals. (**B**) Relative mRNA expression of CD11b and CD14 surface differentiation markers in HL-60-sh-doxy and sh-doxy-YY1 cells untreated and treated with 1 μM ATRA over 96 h at 24 h intervals. (**C**) Relative mRNA expression of CD11b and CD14 surface differentiation markers in OCI-AML3-siSc-CT and siYY1 cells untreated and treated with 1 μM ATRA for 96 h. * *p*-value ≤ 0.05, ** *p*-value ≤ 0.01.

We also assessed CD11b and CD14 expression with qRT-PCR. CD11b was significantly higher in ATRA-untreated HL-60-sh-YY1-doxy cells than in ATRA-untreated HL-60-sh-doxy cells (Figure 8A; CD11b data are graphed separately, as the relative increase in CD11b mRNA in ATRA-treated samples was so much higher than in untreated cells), although no significant increase in CD11b was observed on flow cytometry for the same time points. 

The exposure to ATRA potently induced CD11b in HL-60-sh-doxy, as expected, and the YY1 knockdown enhanced this effect (Figure 8B). CD14 appeared to be significantly increased in the ATRA-untreated HL-60-sh-YY1-doxy cells at 96 h, despite negative flow cytometry, and in the ATRA-treated HL-60-sh-YY1-doxy cells at all time points, compared with the control HL-60-sh-doxy cells. Thus, YY1 knockdown also enhanced CD14 expression in ATRA-treated cells (Figure 8C). 

**Figure 9:**
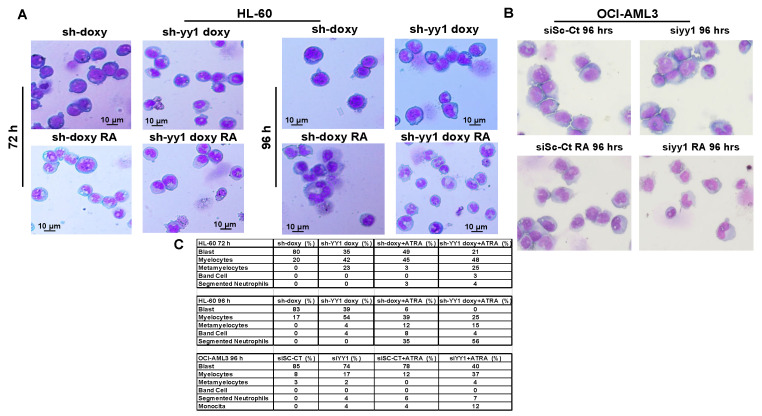
YY1 knockdown induces differentiation morphology in 1 μM ATRA-treated or -untreated HL-60 and OCI-AML3 cells with YY1 knockdown. (**A**) Wright-Giemsa staining of HL-60-sh-doxy and sh-YY1-doxy cells treated and untreated with 1 μM ATRA for 72 h and 96 h. (**B**) Wright-Giemsa staining of siSc-CT and siYY1 cells treated or untreated with 1 μM ATRA for 96 h. (**C**) Table showing the percentage of blasts, myelocytes, metamyelocytes, band cells, and segmented neutrophils in HL-60-sh-doxy and sh-YY1 doxy, in OCI-AML3-siSC-CT and siYY1 cells treated or untreated with ATRA 1μM at indicated time points (50 cells analyzed per field).

Finally, cell morphology as assessed with Wright-Giemsa staining and quantitation of the different cell types, showed that in both HL-60 and OCI-AML3, YY1 knockdown was sufficient to induce chromatin condensation with nuclear segmentation, increased granulation, and reduced cytosolic basophilia (Figure 9A,B). In addition, YY1 knockdown enhanced the pro-differentiation effect of ATRA in both HL-60 and OCI-AML3 cell lines, with a surprising effect in HL-60 cells at 96 h (Figure 9A–C).

Collectively, these data demonstrate that YY1 knockdown overcame the differentiation block, driving HL-60 and OCI-AML3 cells towards granulo/mono cell differentiation. In addition, the YY1 knockdown cells seemed more sensitive to ATRA treatment. Overall, these data demonstrate that YY1 downregulation is not only essential for differentiation but also sensitizes AML cells to the therapeutic effect of ATRA.

### 3.6. YY1 Knockdown Reduces Cell Proliferation and Promotes Apoptosis of AML Cell Lines Untreated and Treated with ATRA

**Figure 10:**
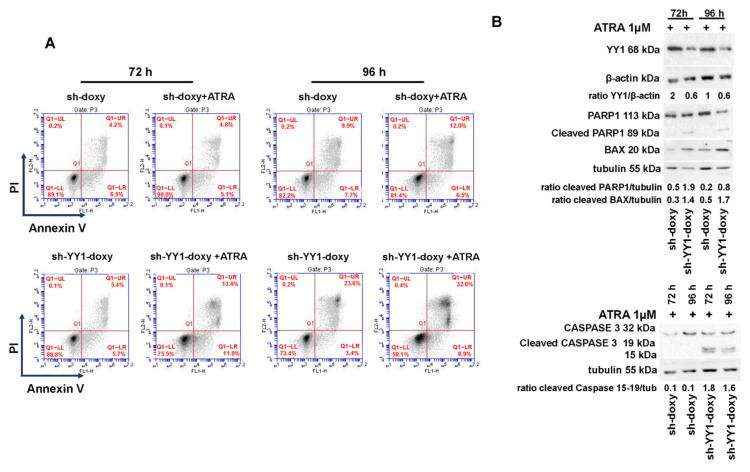
YY1 knockdown induces apoptosis in HL-60 cells. (**A**) Flow cytometry plots showing Annexin V and propidium iodide (PI) double staining in HL-60-sh-doxy (control) and sh-doxy-YY1 cells untreated and treated with 1 μM ATRA for 72 h and 96 h. (**B**) Western blots detecting PARP1, BAX, and caspase 3 protein levels in HL-60-sh-doxy (control) and HL-60-YY1 sh-YY1-doxy cells.

YY1 knockdown reduced cell proliferation of HL-60-sh-YY1-doxy and OCI-AML3-siYY1 cells (Supplementary Figure S3); in addition, several studies have shown that YY1 upregulation inhibits apoptosis and favors growth and cell proliferation in both solid tumors and hematological malignancies [108]. To investigate the effect of YY1 downregulation on cell apoptosis, we assessed Annexin V and PI staining in ATRA-treated and -untreated HL-60-sh-doxy and -sh-YY1-doxy cells. YY1 knockdown per se increased the percentage of Annexin V- and PI-positive cells in HL-60 cells (23% vs. 9.9%, HL-60-sh-YY1-doxy vs. HL-60-sh-doxy at 96 h) (Figure 10A). ATRA treatment did not significantly enhance apoptosis in the HL-60-sh-doxy control cells. However, the percentage of apoptotic/dead ATRA-treated HL-60-sh-YY1-doxy cells was significantly increased relative to control ATRA-treated HL-60-sh-doxy cells (13.4% vs. 4.9% and 32.6% vs. 12%, HL-60-sh-YY1-doxy vs. HL-60-sh-doxy, at 72 h and 96 h, respectively; Figure 10A). Furthermore, we assessed the protein levels of the apoptotic markers caspase 3 and PARP1 and the pro-apoptotic factor BAX in HL-60-sh-YY1-doxy and -sh-doxy cells (Figure 10B). 

The cleaved forms of caspase 3 and PARP1 appeared at 72 h of ATRA treatment in the HL-60-sh-YY1-doxy cells but were absent in control cells, suggesting the activation of terminal apoptosis. In addition, BAX protein levels were also increased in the HL-60-sh-YY1-doxy cells relative to control HL-60-sh-doxy cells at 72 h and 96 h of ATRA treatment (Figure 10B). 

**Figure 11:**
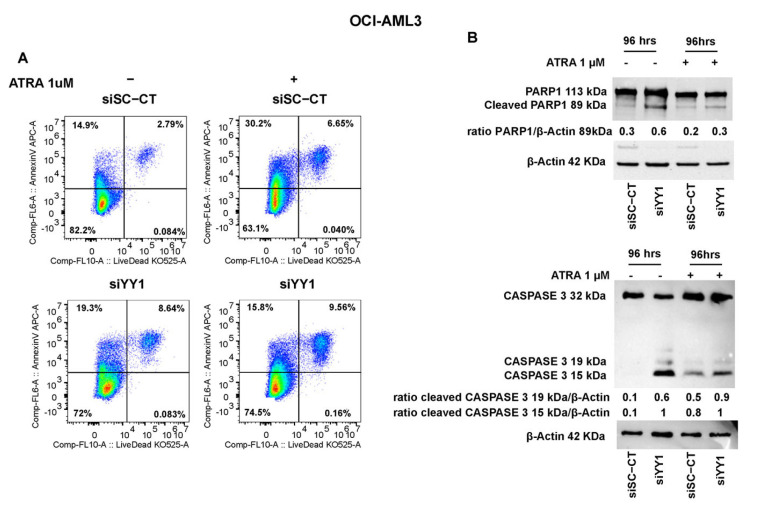
YY1 knockdown induces apoptosis in OCI-AML3 cells. (**A**) Flow cytometry plots showing Annexin V and Live/Dead double staining in OCI-AML3-siSc-CT (control) and siYY1 cells untreated and treated with 1 μM ATRA for 96 h. (**B**) Western blots detecting PARP1 and caspase 3 protein levels in OCI-AML3 siSc-CT (control) and siYY1 cells untreated and treated with ATRA 1 μM for 96 h.

YY1 knockdown also triggered an increase in Annexin V-positive and Live/Dead staining of ATRA-untreated OCI-AML3-siYY1 compared with control OCI-AML3-siSC-CT cells (27.94% vs. 17.69%; Figure 11A). However, the percentage of OCI-AML3-siYY1 Annexin V-positive cells compared with control OCI-AML3-siSC-CT was reduced when these cells were exposed to ATRA, although there was a slight increase in double stained OCI-AML3-siYY1 cells (Annexin V/Dead Live) compared with OCI-AML3-siSC-CT cells (9.56% vs. 6.65%, OCI-AML3-siYY1 vs. OCI-AML3-siSC-CT; Figure 11A). 

PARP1 activation and caspase 3 showed the same trend on Western blots. PARP1 and caspase 3 cleaved forms were more abundant in ATRA-untreated and -treated OCI-AML3-siYY1 cells compared with control OCI-AML3-siSC-CT cells (Figure 11B). 

In conclusion, these data suggest that YY1 downregulation might not only stimulate commitment in HL-60 and OCI-AML3 cells, but also promote apoptosis in these cells. 

## 4. Discussion

The role of the YY1 protein in carcinogenesis and the progression of solid tumors is well established [76,77,78,79,80,81,82,83,84,85], as well as in lymphoid leukemia [109,110,111,112]. Although YY1 is generally overexpressed in AML [88,89], it is not known if YY1 overexpression may, per se, induce leukemic transformation. In addition, data from the cancer genome atlas (TCGA) database (https://servers.binf.ku.dk/bloodspot/, accessed on 1 July 2023) highlighted a heterogenous YY1 expression among AML samples, without the presence of associations with specific mutational features and patient prognosis (Supplementary Figure S4). However, to date, no molecular studies have been conducted to determine the mechanisms by which YY1 may interfere with normal myelopoiesis, and above all, no data are available on the effect of YY1 downregulation in human AML cells. In this study, we knocked down YY1 in human AML cells, primarily through RNA interference strategies, to investigate the role of YY1 in myeloid differentiation and AML. YY1 knockdown in an AML sample and AML cell lines restored/increased mRNA and protein expression of C/EBP family members, including C/EBPα, C/EBPε, and C/EBPδ, involved in normal and leukemic myelopoiesis [113], and promoted the expression of pro-myeloid growth factor receptors such as GM-CSFr and G-CSFr in HL-60 and of GM-CSFr in OCI-AML3. YY1 knockdown also increased the sensitivity of an AML patient sample and the AML cell lines to ATRA treatment, a standard-of-care therapy used in the clinic. ChIp experiments revealed that YY1 occupied C/EBPα, C/EBPε, and C/EBPδ 5′ promoter regions in the AML cell lines, thus functioning in this context as a suppressor of transcription. Finally, we showed that YY1 is able to bind the RARα promoter region, the ATRA receptor, and that the loss of YY1 increased the expression of RARα per se. Thus, an explanation for the increased sensitivity of AML cells with YY1 knockdown to ATRA may be related to the removal of the repressive effect on RARα transcription and to the increased availability of the ATRA receptor [114].

Remarkably, YY1 knockdown not only induced the expression of C/EBPα, C/EBPε, C/EBPδ, and RARα but also relieved the differentiation block in AML cells. In HL-60 cells, YY1 downregulation generated cell populations that showed robust expression of CD11b but nearly negative CD14 staining. ATRA exposure boosted the expression of CD11b and a significant increase in CD14. In OCI-AML3, YY1 downregulation produced the same results, even if quantitatively less important. This result is probably related to the fact that the cell lines are at a different stage of differentiation block (OCI-AML3 is a FAB M4 acute myelomonocytic leukemia, and HL-60 is a FAB M2 acute myeloblastic leukemia with maturation). These data were supported by the morphological analyses. YY1 knockdown in HL-60 and OCI-AML3 cells clearly showed a recovery of commitment toward mature myeloid lineages, and this direction towards differentiation was even more evident when cells were exposed to ATRA. In particular, ATRA-treated HL-60-sh-YY1-doxy cells appeared to achieve a high degree of granulocytic differentiation. Our data paralleled the morphologic studies conducted by Erkeland et al. [89], showing an impairment of myeloid commitment when YY1 was ectopically expressed in a murine hematopoietic progenitor cell line model. 

Evasion from apoptosis is a hallmark of malignant tumor progression, and identifying strategies to induce or restore defective apoptosis is a major priority in the development of cancer therapy. The development of AML has also been shown to be dependent on dysregulation of the apoptotic pathway. For instance, the overexpression of BCL2, which is an important antiapoptotic protein in AML, led to the development of BCL2 inhibitors to promote the induction of apoptosis in AML cells and has led to the discovery of venetoclax, a potent and selective BCL2 inhibitor [115]. More than 250 scientific articles (PubMed) show a direct role for the deregulated expression of YY1 in inhibiting apoptotic pathways through a variety of mechanisms. For the first time, our data show that the reduction in YY1 expression in HL-60 and OCI-AML3 cell lines restored apoptosis as shown by the activation of PARP1 and caspase 3 and the increased expression of the pro-apoptotic protein BAX. 

## 5. Conclusions

Collectively, our data propose a central role for YY1 in the development of AML. YY1 downregulation restored the expression of myeloid C/EBP transcription factors and growth factors; increased the availability of RARα, making cells more sensitive to ATRA exposure; and restored apoptosis in AML cell lines. Therefore, YY1 represents a novel target of investigation in the quest to improve AML patient treatment [39].

## Data Availability

Data supporting the reported results are maintained in a research folder in the Department of Experimental Medicine, Epigenetic Laboratory Z.G., University of Rome “Sapienza”.

## Supplementary Materials

The following supporting information can be downloaded at: https://www.mdpi.com/article/10.3390/cancers15154010/s1, File S1: The original western blot figures; Figure S1: mRNA expression of myeloid growth factors and receptors in HL-60 and OCI-AML3 YY1 interfered cell lines. mRNA expression levels were normalized to GAPDH levels and expressed as fold induction to control cells value (=1). Each qRT-PCR amplification was performed in triplicate. Statistical analysis was performed in triplicate using the nonparametric Mann-Whitney test. * *p* value ≤ 0.05. Error bars: SD, standard deviation; Figure S2: mRNA expression of myeloid growth factors and receptors in HL-60 control sh-doxy and YY1 interfered sh-yy1-doxy cell lines. mRNA expression levels were normalized to GAPDH levels and expressed as fold induction to control cells value (=1). Each qRT-PCR amplification was performed in triplicate. Statistical analysis was performed in triplicate using the nonparametric Mann-Whitney test. * *p* value ≤ 0.05. Error bars: SD, standard deviation; Figure S3: Cell proliferation of YY1 knocked down HL-60 (sh-yy1 doxy) and OCI-AML3 (siyy1) cells vs. their respective cell controls HL-60 sh-doxy and OCI-AML3 siSC-CT, at the indicated time points. * *p* value ≤ 0.05; Figure S4: data from the cancer genome atlas (TCGA) database (https://servers.binf.ku.dk/bloodspot/) highlighted a heterogenous YY1 expression among AML samples, without the presence of associations with specific mutational features and patient prognosis; Table S1: AML patient sample characteristics.
