# Supplementary material for: YY1 Knockdown Relieves the Differentiation Block and Restores Apoptosis in AML Cells
Source: Cancers (Basel). 2023 Aug 7;15(15):4010. doi: 10.3390/cancers15154010 (PMC10417667; doi:10.3390/cancers15154010)
Supplement: Supplementary file 1 [file cancers-15-04010-s001.zip › cancers-2486923-File S1.pdf]

Figure 1

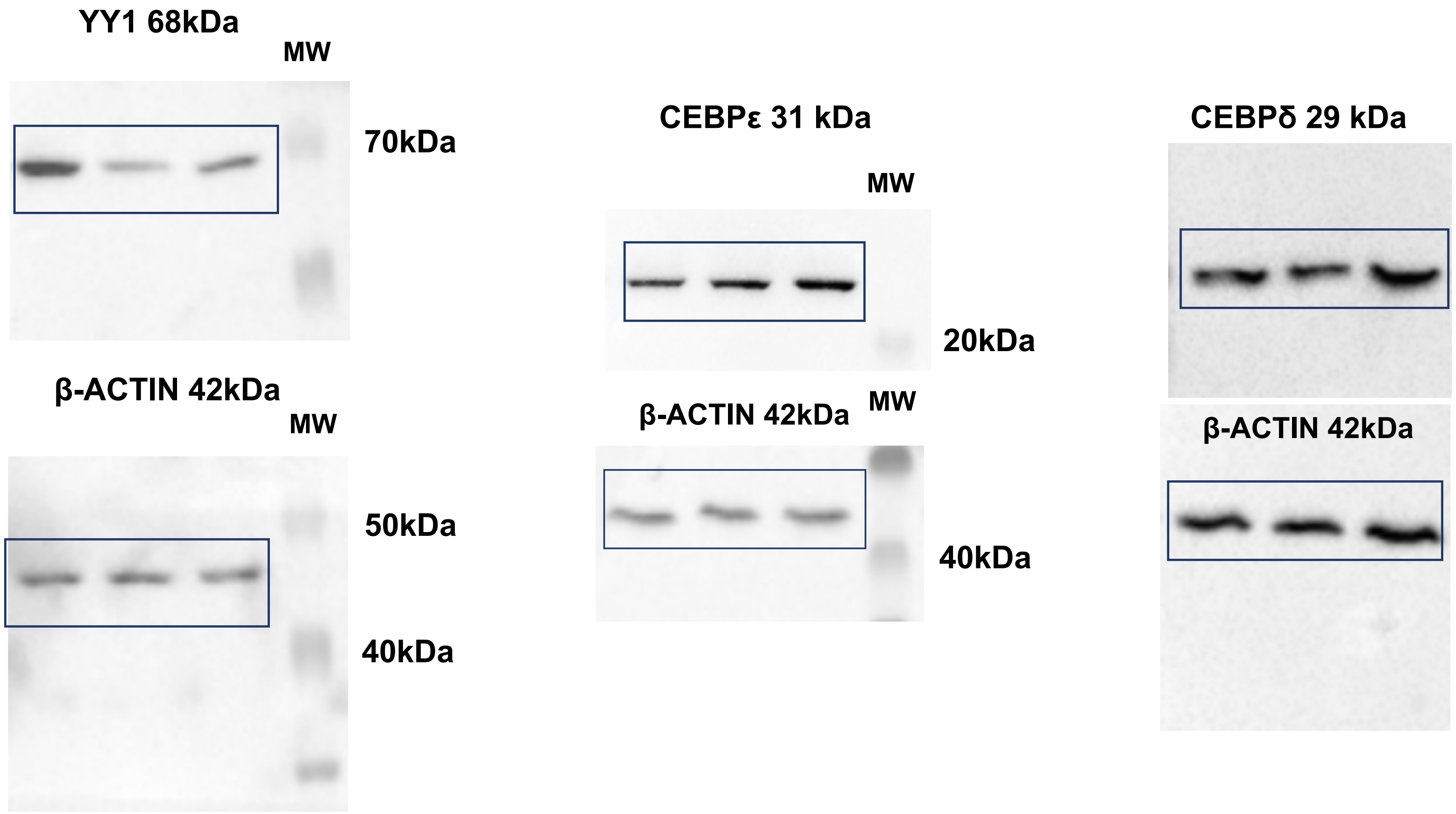
YY1 68kDa
MW
70kDa
β-ACTIN 42kDa
MW
50kDa
40kDa
CEBPε 31 kDa
MW
20kDa
β-ACTIN 42kDa
MW
40kDa
CEBPδ 29 kDa
β-ACTIN 42kDa

Figure 2A

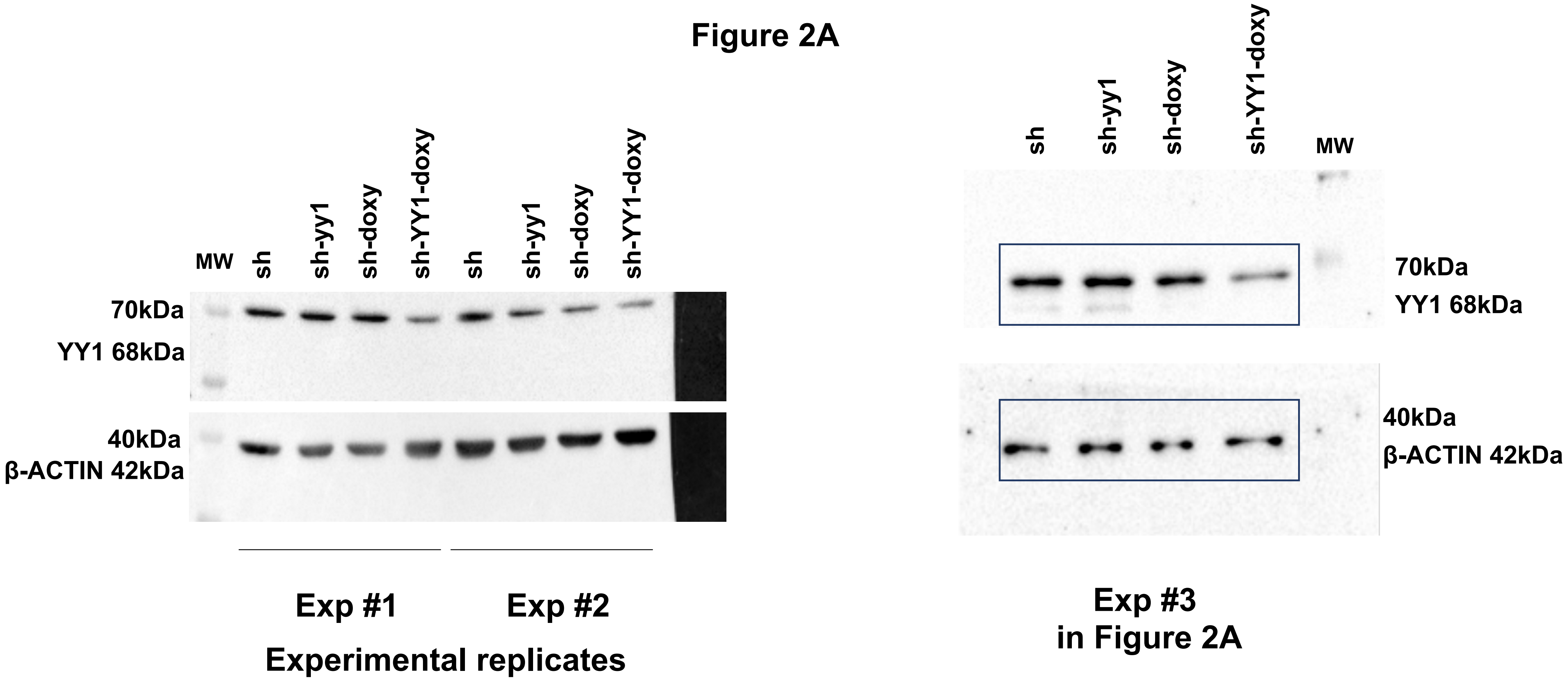

Exp #3
in Figure 2A

Figure 2C

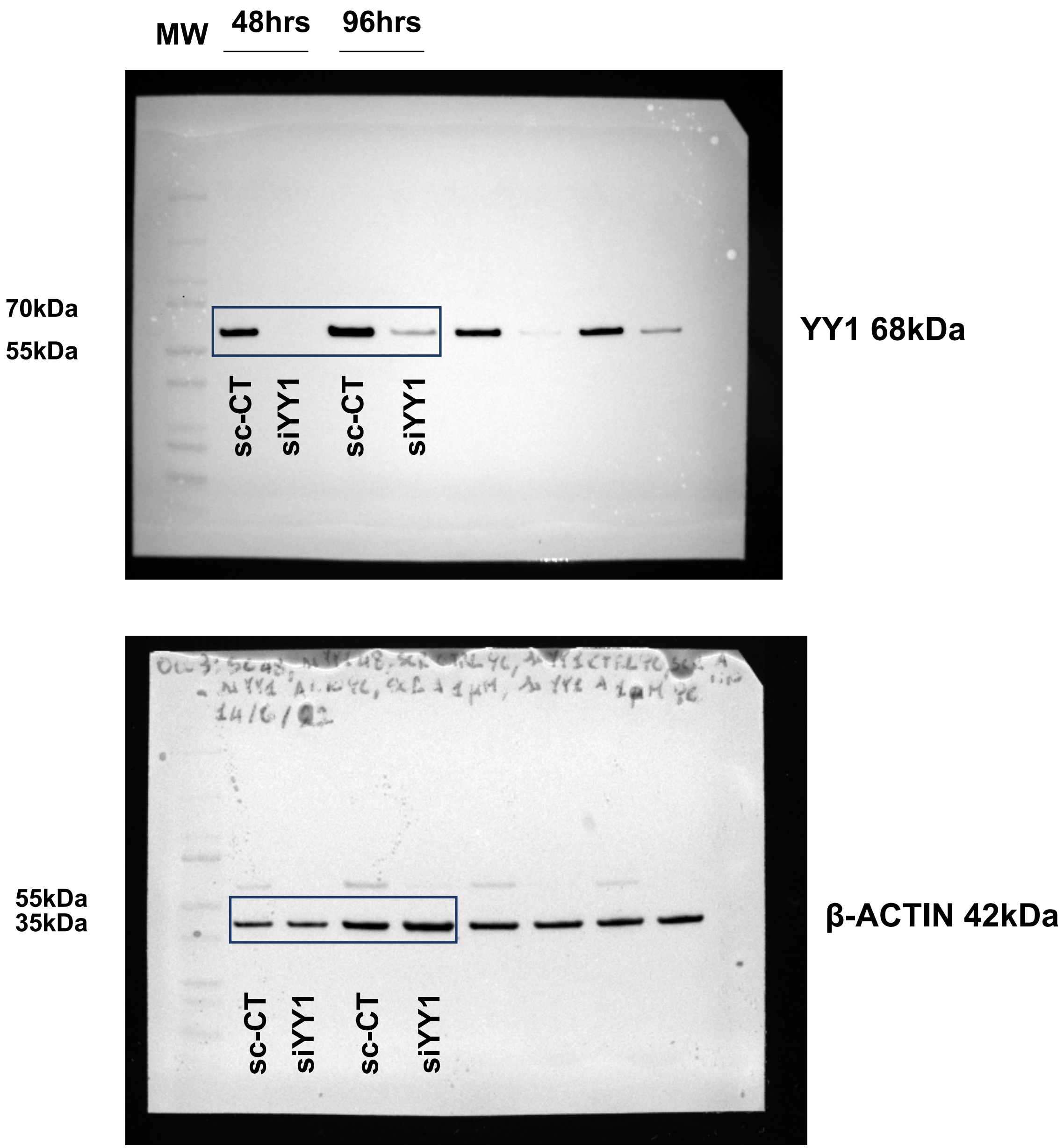

MW
48hrs
96hrs
70kDa
55kDa
sc-CT
siYY1
sc-CT
siYY1
YY1 68kDa
55kDa
35kDa
sc-CT
siYY1
sc-CT
siYY1
β-ACTIN 42kDa

Figure 3

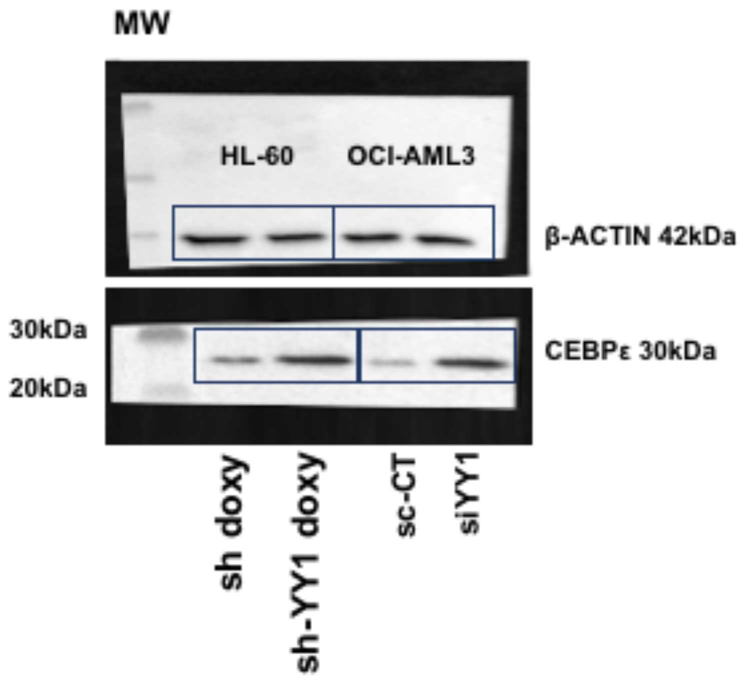
MW
HL-60
OCI-AML3
β-ACTIN 42kDa
30kDa
20kDa
CEBPε 30kDa
sh doxy
sh-YY1 doxy
sc-CT
siYY1

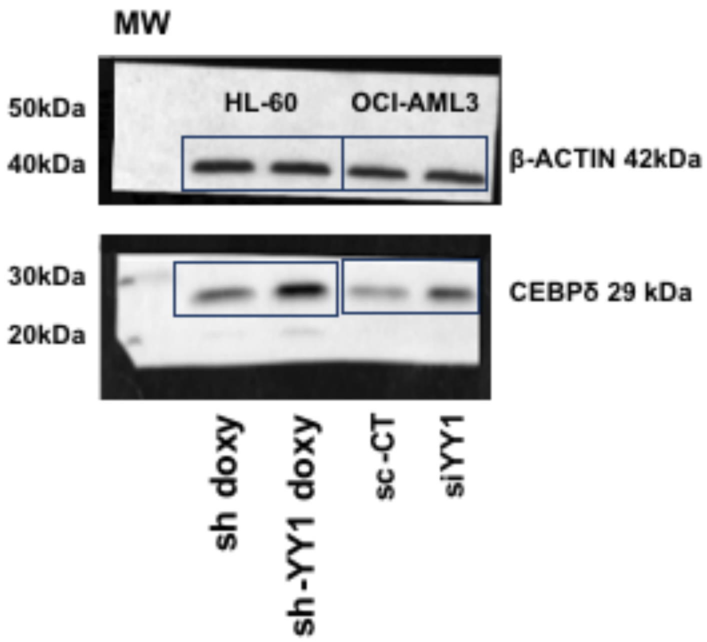
MW
50kDa
40kDa
HL-60
OCI-AML3
β-ACTIN 42kDa
30kDa
20kDa
CEBPδ 29 kDa
sh doxy
sh-YY1 doxy
sc-CT
siYY1

Figure 4

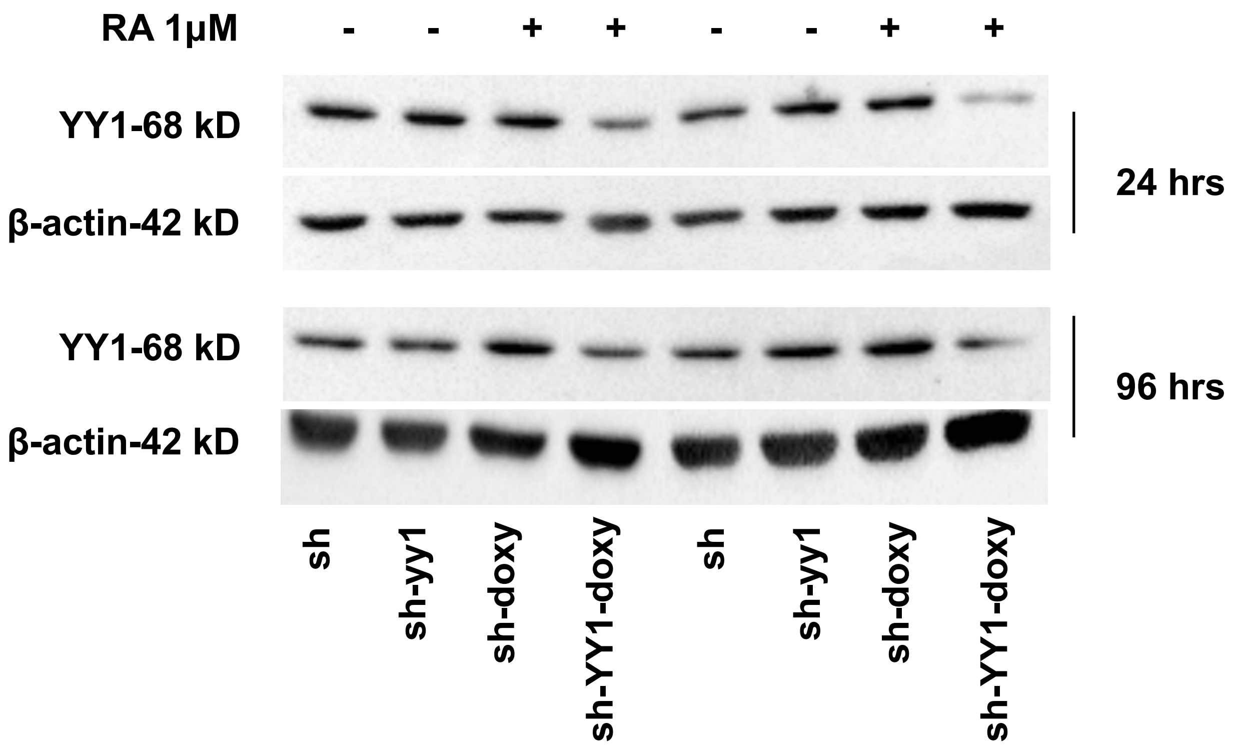
RA 1µM
- - + + - - + +
YY1-68 kD
β-actin-42 kD
24 hrs
YY1-68 kD
β-actin-42 kD
96 hrs
sh
sh-yy1
sh-doxy
sh-YY1-doxy
sh
sh-yy1
sh-doxy
sh-YY1-doxy

Figure 5

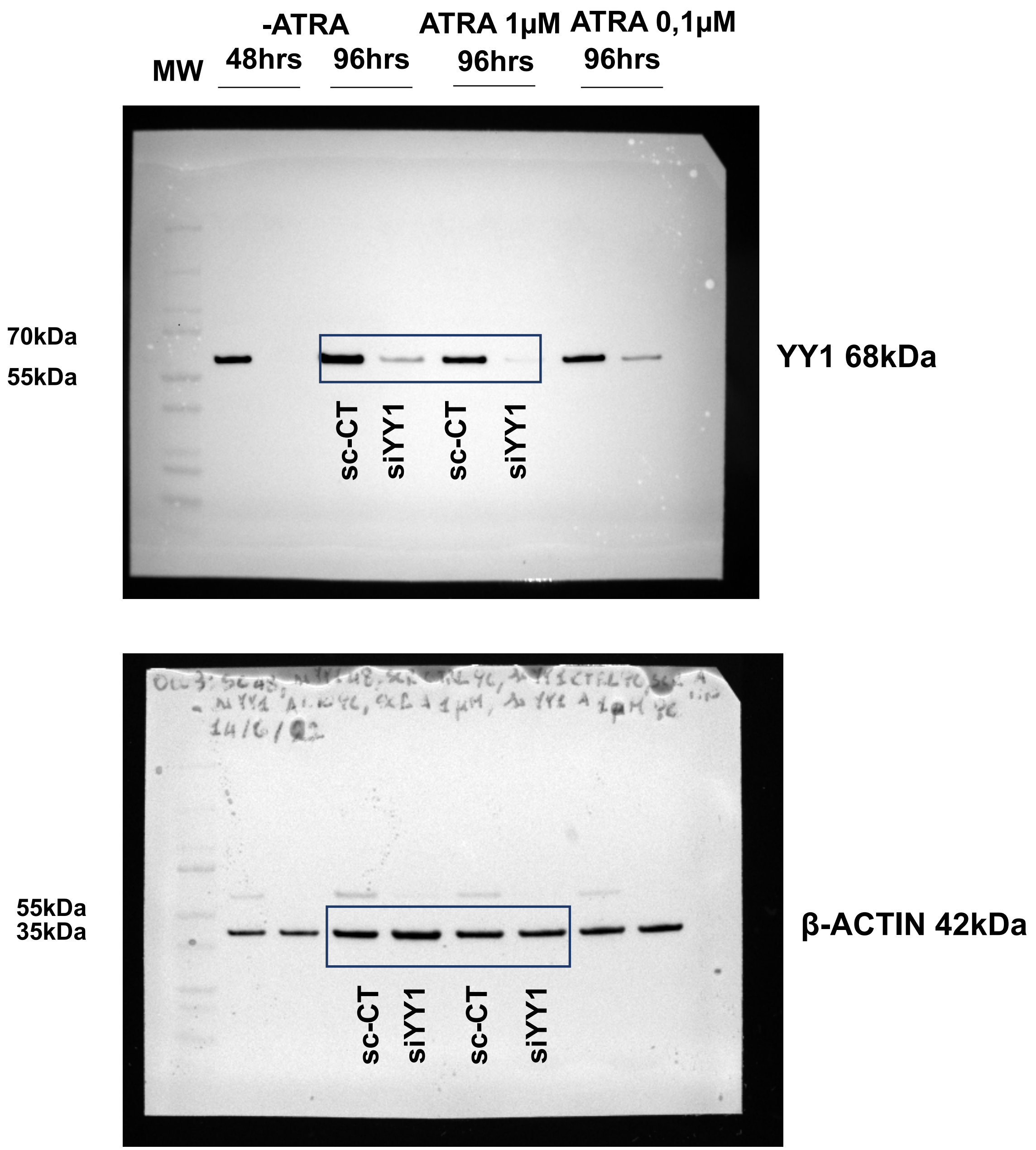
MW
-ATRA
48hrs
96hrs
ATRA 1µM
96hrs
ATRA 0,1µM
96hrs
70kDa
55kDa
YY1 68kDa
sc-CT
siYY1
sc-CT
siYY1
55kDa
35kDa
β-ACTIN 42kDa
sc-CT
siYY1
sc-CT
siYY1

FIGURE 10

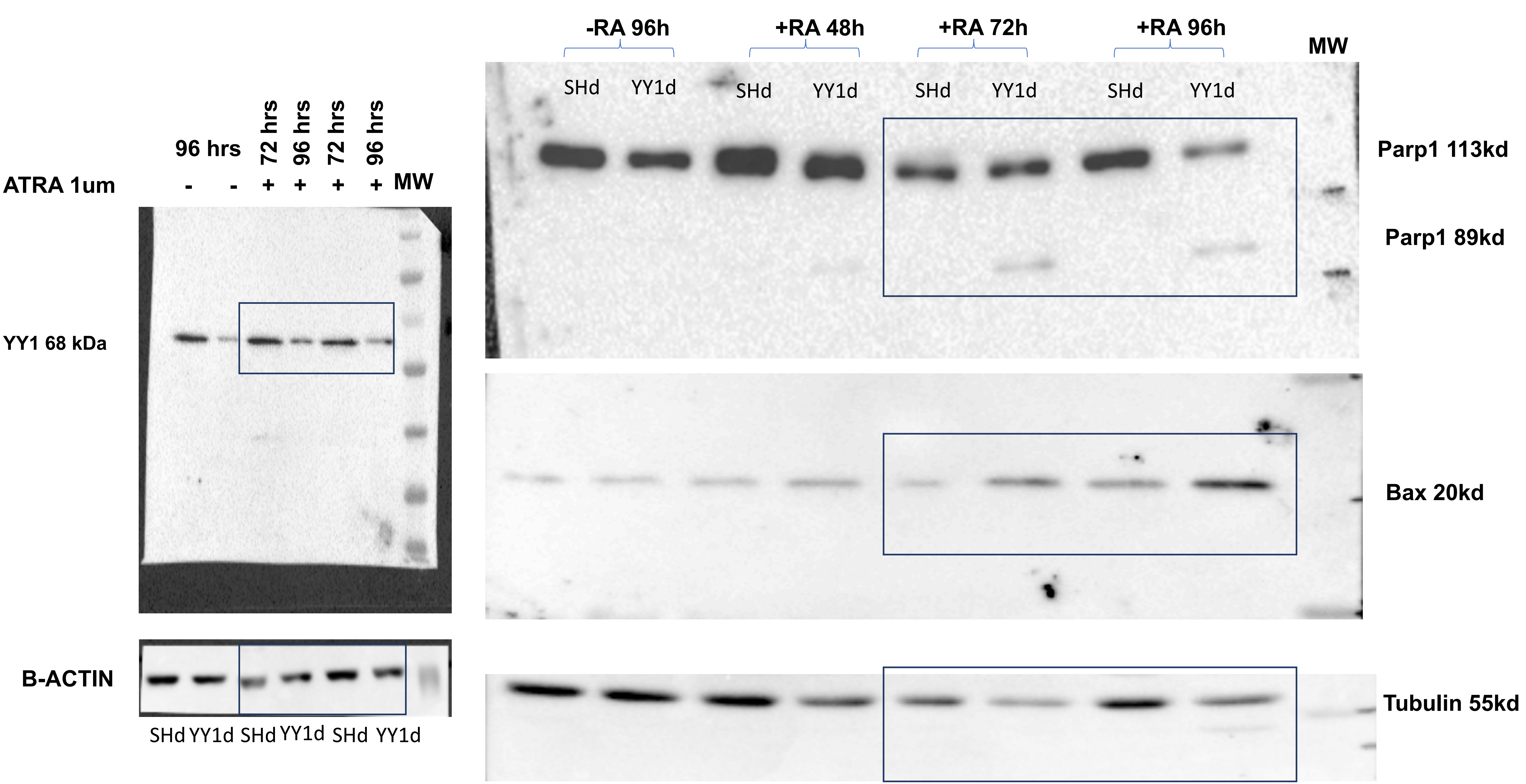
96 hrs
72 hrs
96 hrs
72 hrs
96 hrs
ATRA 1um
- - + + + + MW
YY1 68 kDa
B-ACTIN
SHd YY1d SHd YY1d SHd YY1d
-RA 96h
+RA 48h
+RA 72h
+RA 96h
MW
SHd YY1d SHd YY1d SHd YY1d SHd YY1d
Parp1 113kd
Parp1 89kd
Bax 20kd
Tubulin 55kd

FIGURE 10

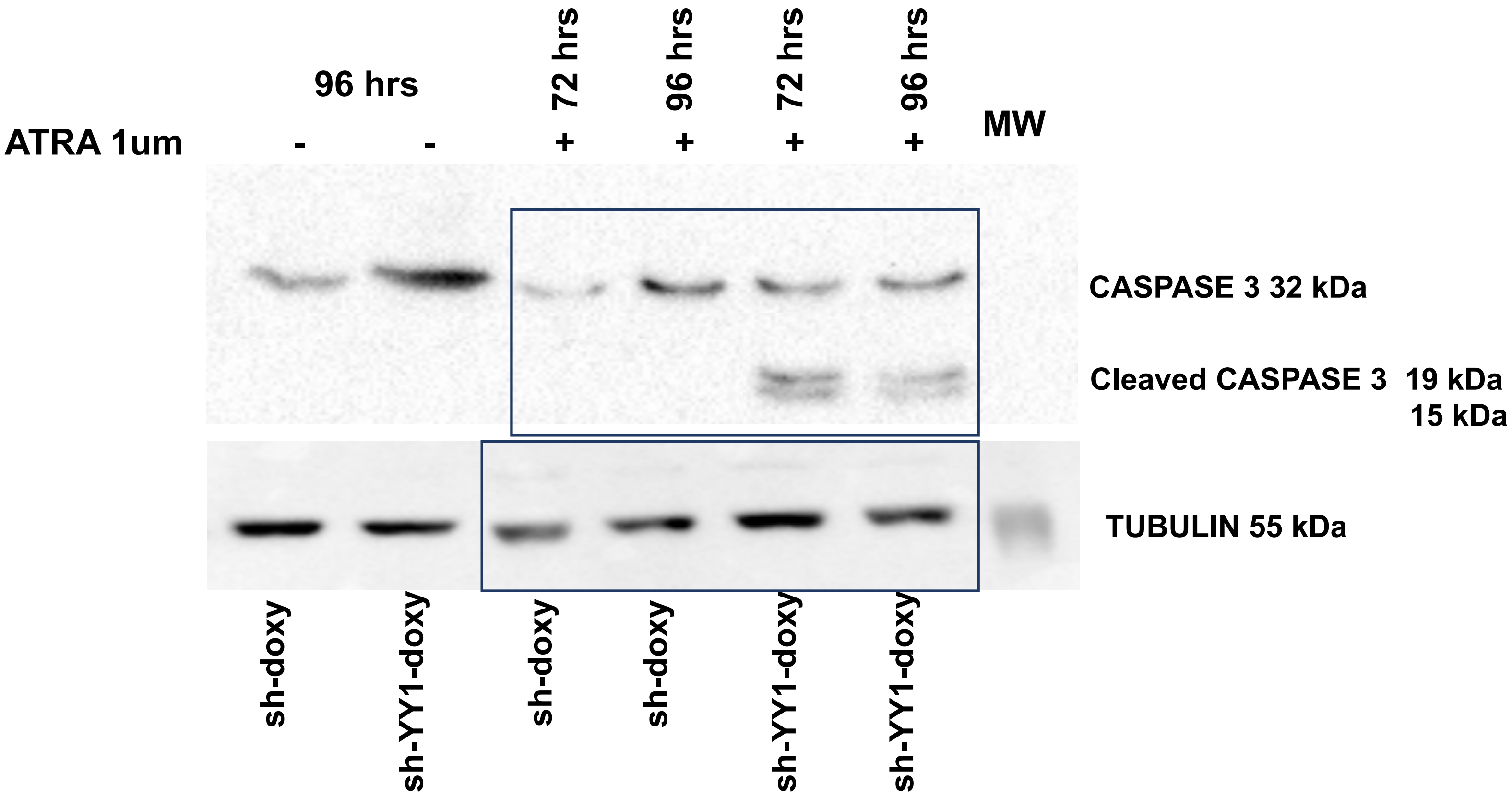
96 hrs
72 hrs
96 hrs
72 hrs
96 hrs
ATRA 1um
- - + + + +
MW
CASPASE 3 32 kDa
Cleaved CASPASE 3 19 kDa
15 kDa
TUBULIN 55 kDa
sh-doxy
sh-YY1-doxy
sh-doxy
sh-doxy
sh-YY1-doxy
sh-YY1-doxy

Figure 11

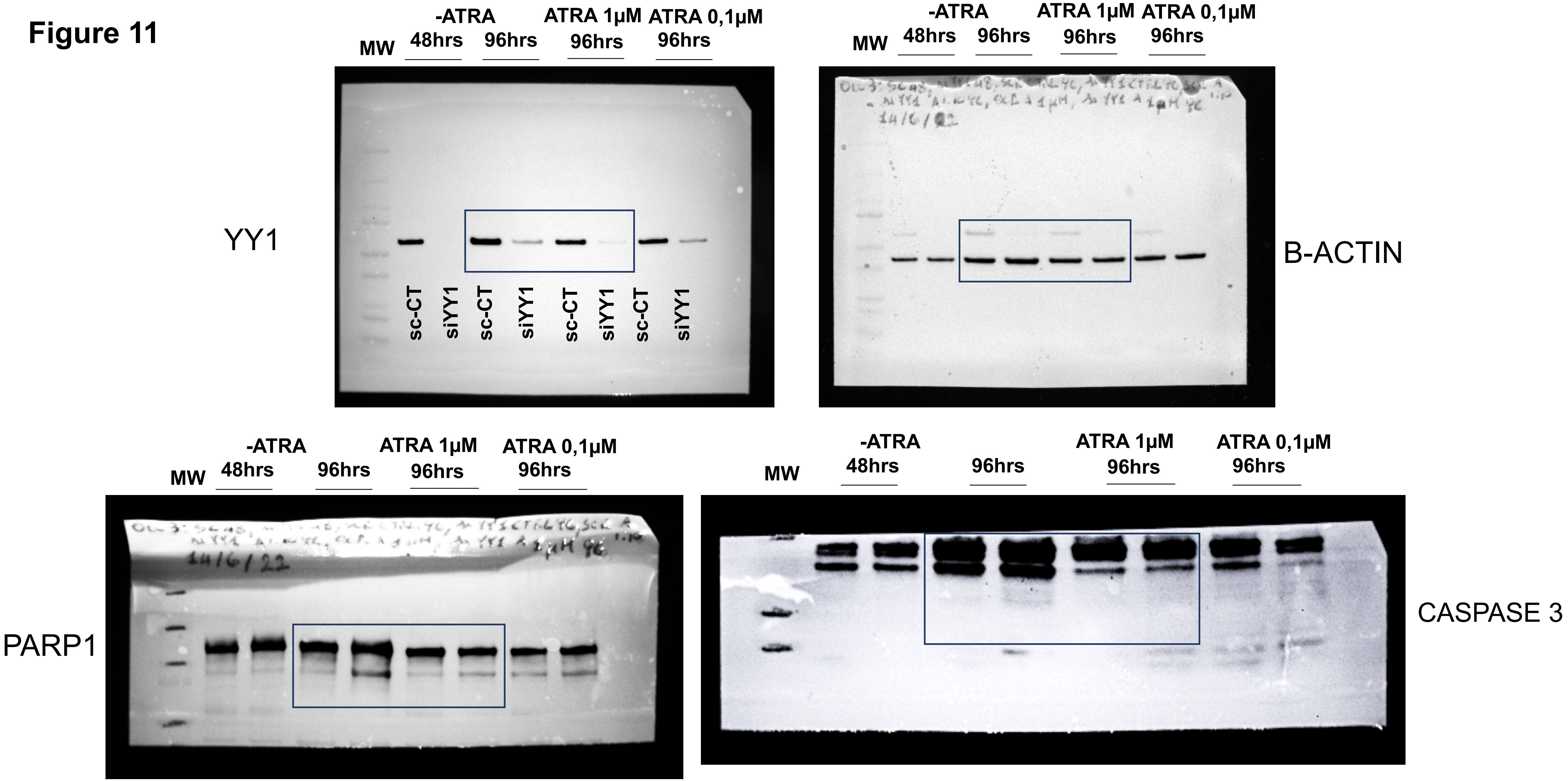

File S1. The original western blot figures.
