# Supplementary material for: YY1 Knockdown Relieves the Differentiation Block and Restores Apoptosis in AML Cells
Source: Cancers (Basel). 2023 Aug 7;15(15):4010. doi: 10.3390/cancers15154010 (PMC10417667; doi:10.3390/cancers15154010)
Supplement: Supplementary file 1 [file cancers-15-04010-s001.zip › cancers-2486923-supplementary figures and table.pdf]

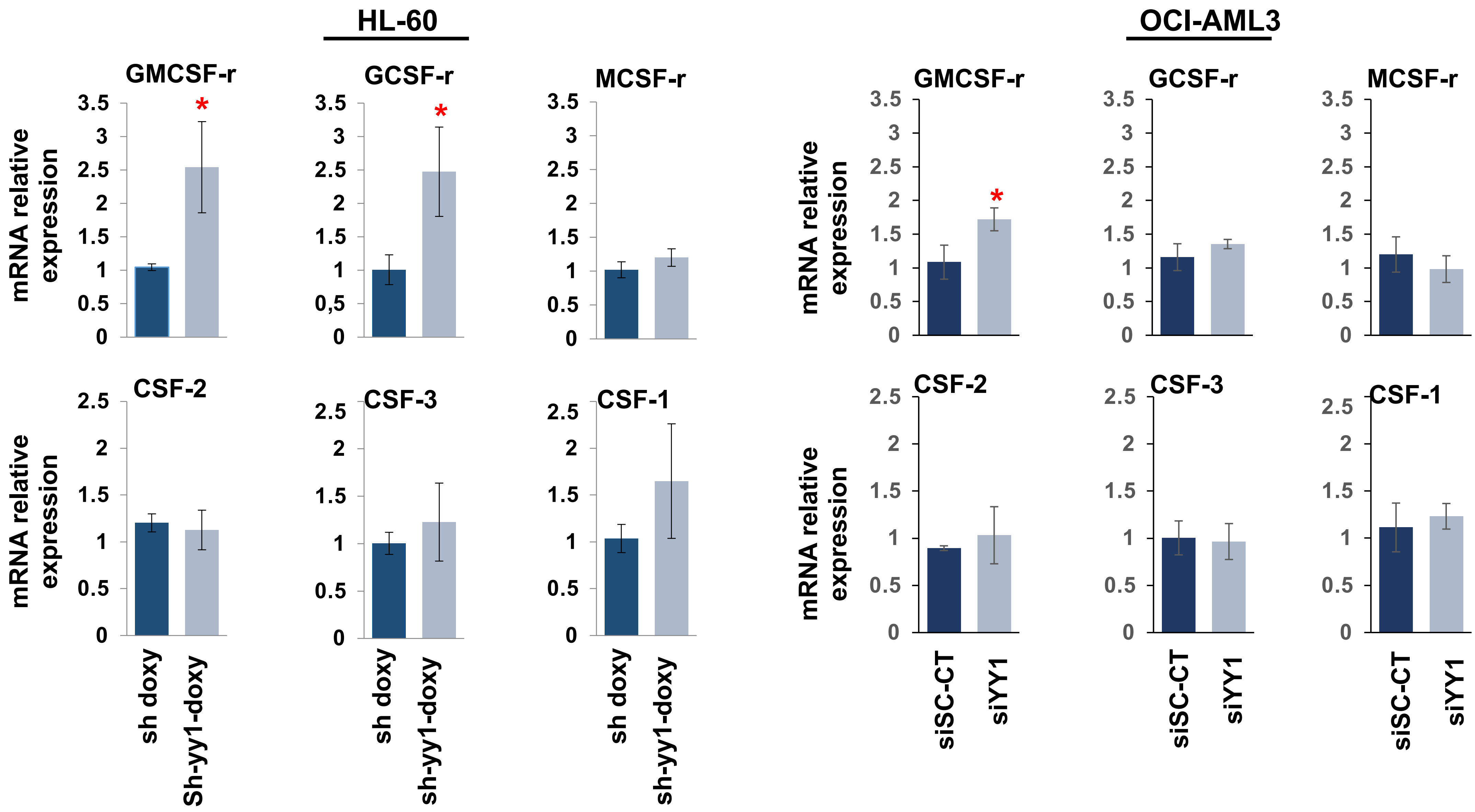

Supplementary Figure S1: mRNA expression of myeloid growth factors and receptors in HL-60 and OCI-AML3 YY1 interfered cell lines. mRNA expression levels were normalized to GAPDH levels and expressed as fold induction to control cells value (=1). Each qRT-PCR amplification was performed in triplicate. Statistical analysis was performed in triplicate using the nonparametric Mann-Whitney test. * p value ≤ 0.05. Error bars: SD, standard deviation.

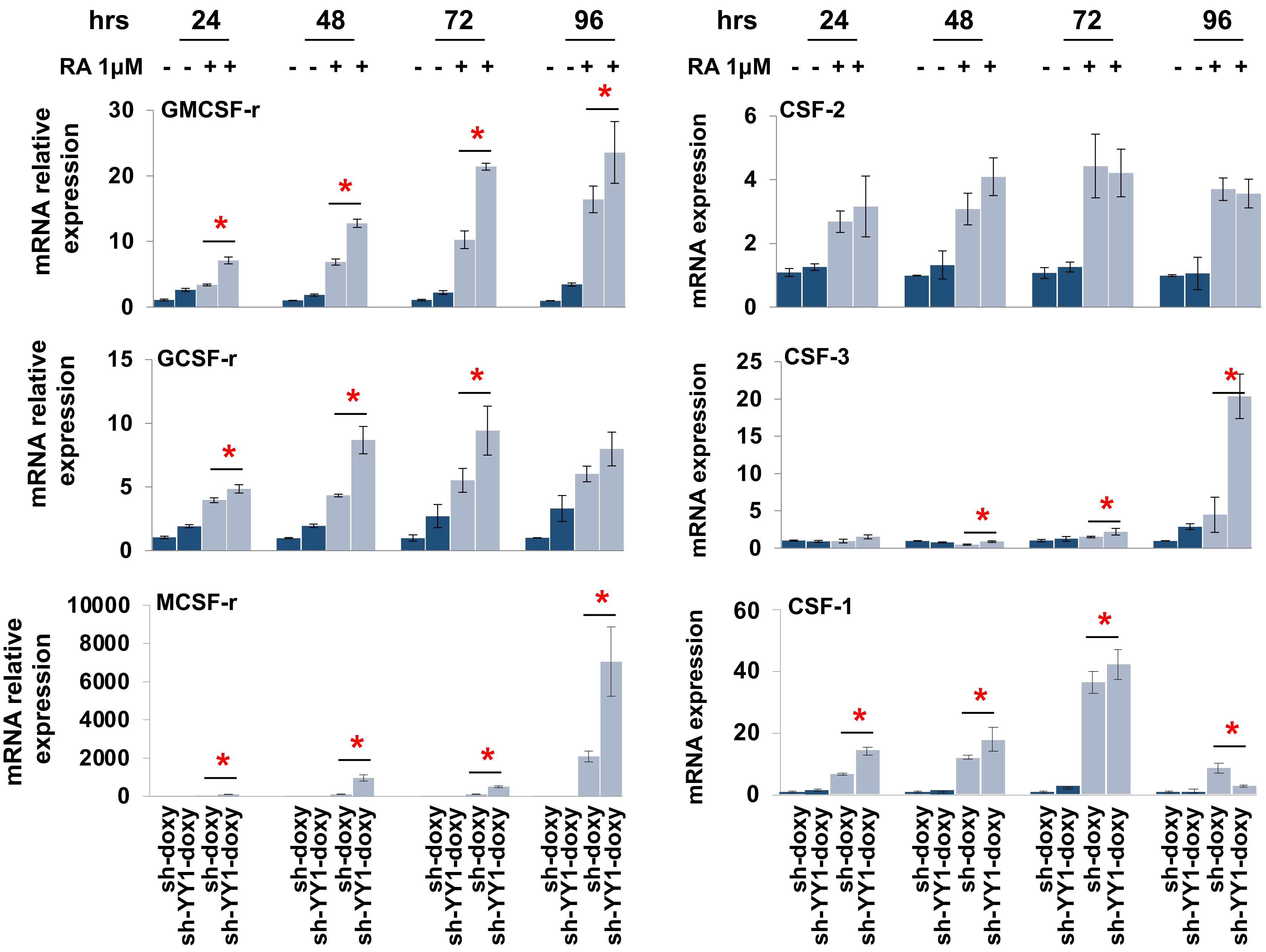

Supplementary Figure S2: mRNA expression of myeloid growth factors and receptors in HL-60 control sh-doxy and YY1 interfered sh-yy1-doxy cell lines. mRNA expression levels were normalized to GAPDH levels and expressed as fold induction to control cells value (=1). Each qRT-PCR amplification was performed in triplicate. Statistical analysis was performed in triplicate using the nonparametric Mann-Whitney test. * p value ≤ 0.05. Error bars: SD, standard deviation.

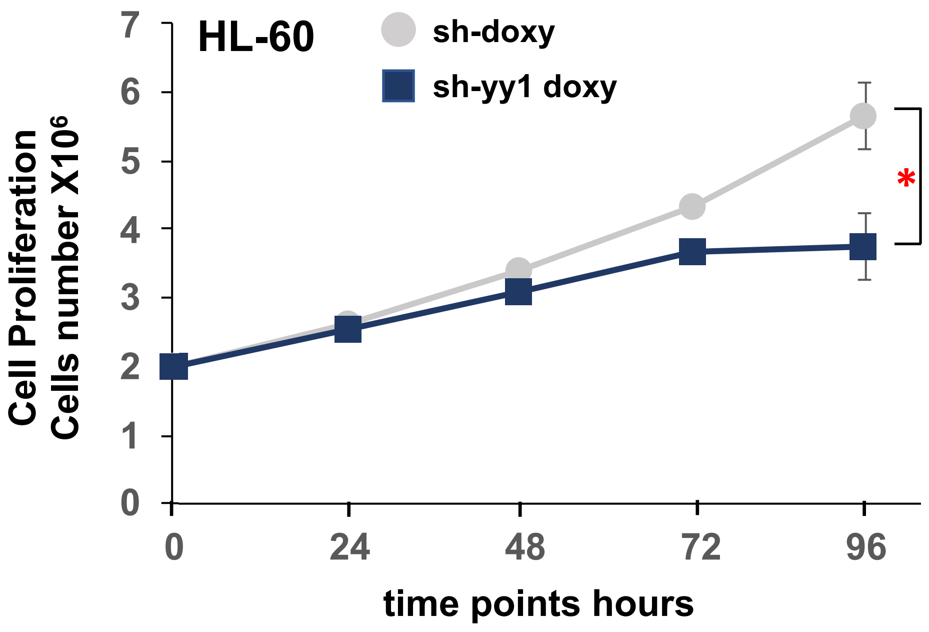

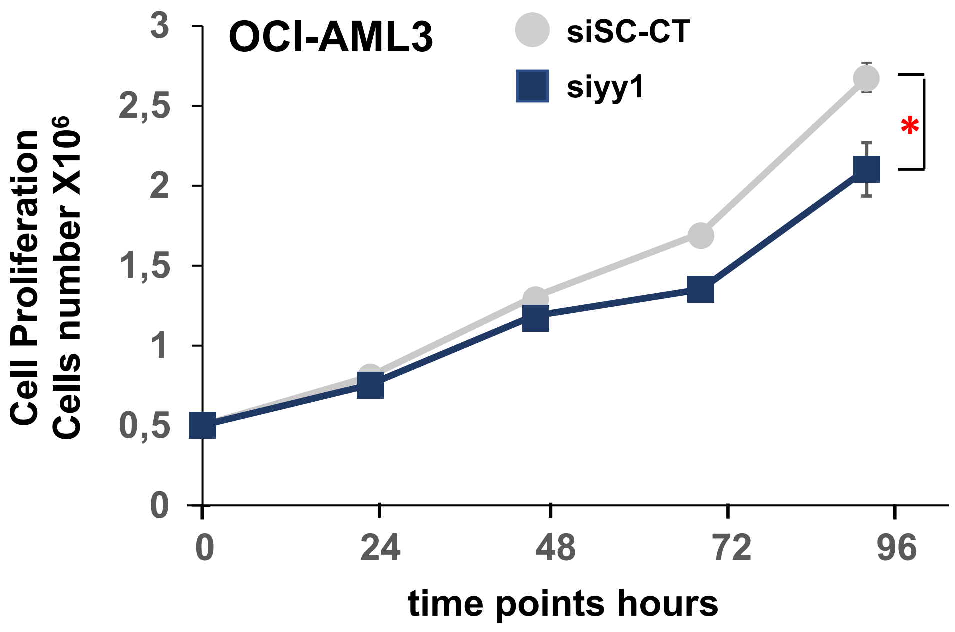

Supplementary Figure S3: Cell proliferation of YY1 knocked down HL-60 (sh-yy1 doxy) and OCI-AML3 (siyy1) cells *vs* their respective cell controls HL-60 sh-doxy and OCI-AML3 siSC-CT, at the indicated time points. * p value ≤ 0.05.

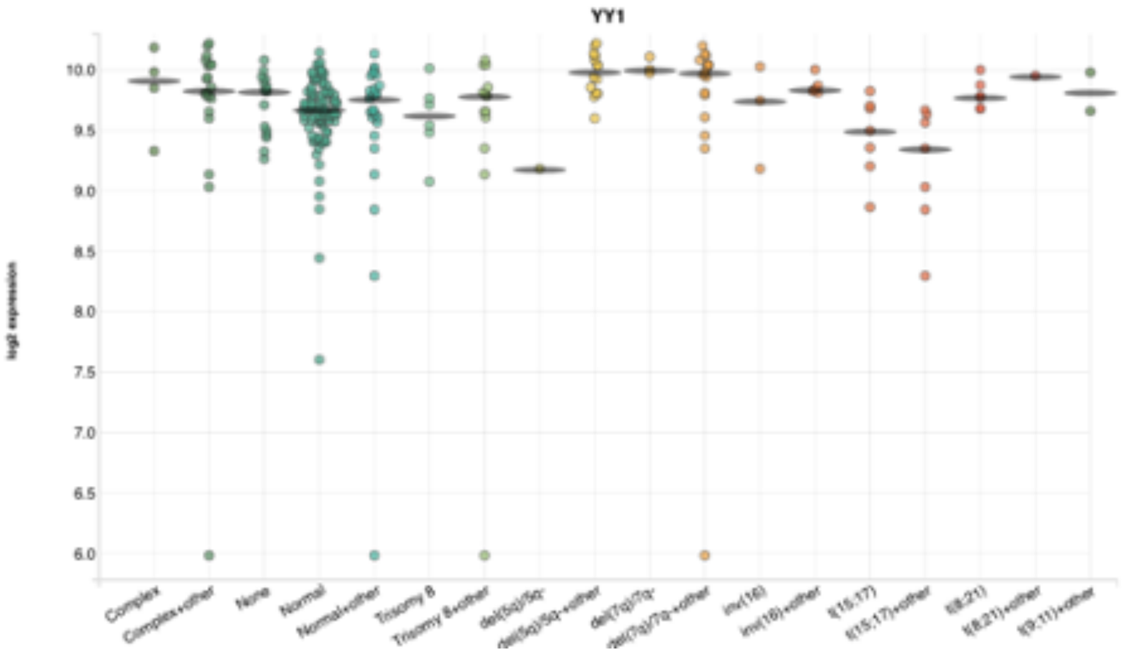

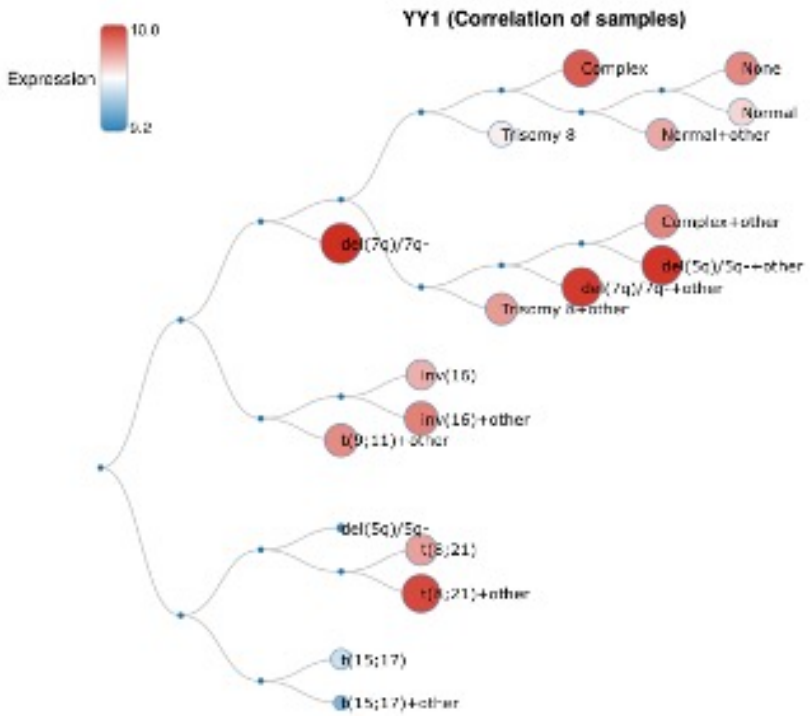

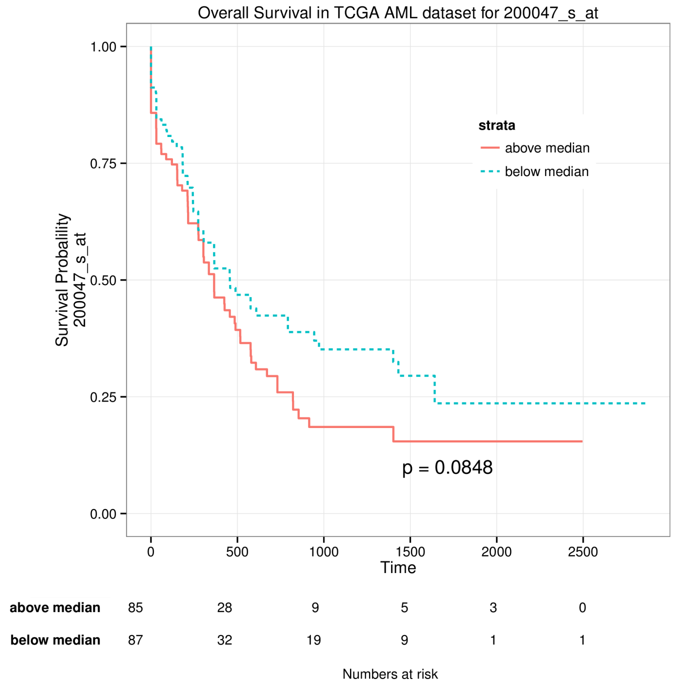

Supplementary Figure S4: data from the cancer genome atlas (TCGA) database (https://servers.binf.ku.dk/bloodspot/) highlighted a heterogenous YY1 expression among AML samples, without the presence of associations with specific mutational features and patient prognosis.

Supplementary Table S1: AML patient sample characteristics.

Patient #	Karyotype	Mutation
1	46, XX	-
2	47, XX, +8	-
3	46, XY	-
4	47, XX, +11	*FLT3*-TKD
5	46, XY	*FLT3*-ITD
6	46, XY	-
7	46, XY	-
8	46, XY	-
9	46, XY	-
10	46, XY	-
11	46, XX	*FLT3*-ITD, *NPM1*
12	-	*FLT3*-ITD, *NPM1*
13	-	-
14	46, XX	*FLT3*-TKD, *NPM1, IDH2*
15	-	*FLT3*-ITD, *NPM1, IDH1*
16	46, XX	*NPM1*
17	46, XY	*FLT3*-ITD
18	46, XY	*FLT3*-TKD
19	-	-
20 relapse	46, XX	-
21	-	*IDH1*
22	-	*FLT3*-TKD, *NPM1*
23	47, XY, +8	*IDH2*
24	46, XY, t(15;17)	-
